# Genetic variants associated with Alzheimer’s disease confer different cerebral cortex cell-type population structure

**DOI:** 10.1186/s13073-018-0551-4

**Published:** 2018-06-08

**Authors:** Zeran Li, Jorge L. Del-Aguila, Umber Dube, John Budde, Rita Martinez, Kathleen Black, Qingli Xiao, Nigel J. Cairns, The Dominantly Inherited Alzheimer Network (DIAN), Joseph D. Dougherty, Jin-Moo Lee, John C. Morris, Randall J. Bateman, Celeste M. Karch, Carlos Cruchaga, Oscar Harari

**Affiliations:** 1 Department of Psychiatry, Washington University School of Medicine, 660 S. Euclid Ave. B8134, St. Louis, MO 63110 USA; 2 Medical Scientist Training Program, Washington University School of Medicine, 660 S. Euclid Ave, St. Louis, MO 63110 USA; 3 Department of Neurology, Washington University School of Medicine, 660 S. Euclid Ave, St. Louis, MO 63110 USA; 4 Department of Pathology & Immunology, Washington University in St. Louis, School of Medicine, 510 S. Kingshighway, MC 8131, Saint Louis, MO 63110 USA; 5 Knight Alzheimer’s Disease Research Center, Washington University School of Medicine, 660 S. Euclid Ave, St. Louis, MO 63110 USA; 6 Hope Center for Neurological Disorders, Washington University School of Medicine, 660 S. Euclid Ave. B8111, St. Louis, MO 63110 USA; 7 Department of Genetics, Washington University School of Medicine, 660 S. Euclid Ave, St. Louis, MO 63110 USA

**Keywords:** Digital deconvolution, Alzheimer’s disease, Brain cellular composition, Bulk RNA-sequencing, Autosomal dominant AD, *TREM2*

## Abstract

**Background:**

Alzheimer’s disease (AD) is characterized by neuronal loss and astrocytosis in the cerebral cortex. However, the specific effects that pathological mutations and coding variants associated with AD have on the cellular composition of the brain are often ignored.

**Methods:**

We developed and optimized a cell-type-specific expression reference panel and employed digital deconvolution methods to determine brain cellular distribution in three independent transcriptomic studies.

**Results:**

We found that neuronal and astrocyte relative proportions differ between healthy and diseased brains and also among AD cases that carry specific genetic risk variants. Brain carriers of pathogenic mutations in *APP*, *PSEN1*, or *PSEN2* presented lower neuron and higher astrocyte relative proportions compared to sporadic AD. Similarly, the *APOE ε*4 allele also showed decreased neuronal and increased astrocyte relative proportions compared to AD non-carriers. In contrast, carriers of variants in *TREM2* risk showed a lower degree of neuronal loss compared to matched AD cases in multiple independent studies.

**Conclusions:**

These findings suggest that genetic risk factors associated with AD etiology have a specific imprinting in the cellular composition of AD brains. Our digital deconvolution reference panel provides an enhanced understanding of the fundamental molecular mechanisms underlying neurodegeneration, enabling the analysis of large bulk RNA-sequencing studies for cell composition and suggests that correcting for the cellular structure when performing transcriptomic analysis will lead to novel insights of AD.

**Electronic supplementary material:**

The online version of this article (10.1186/s13073-018-0551-4) contains supplementary material, which is available to authorized users.

## Background

Alzheimer’s disease (AD) is a neurodegenerative disorder characterized clinically by gradual and progressive memory loss and pathologically by the presence of senile plaques (Aβ deposits) and neurofibrillary tangles (NFTs, Tau deposits) in the brain [1]. AD has a substantial but heterogeneous genetic component. Mutations in the amyloid-beta precursor protein (*APP*) and Presenilin genes (*PSEN1* and *PSEN2*) [2, 3] cause autosomal dominant AD (ADAD) which is typically associated with early-onset (< 65 years). In contrast, the most common manifestation of AD presents late-onset (LOAD) and accounts for the majority of the cases (90–95%). Despite appearing sporadic in nature, a complex genetic architecture underlies LOAD risk. *APOE* ε4 is the most common genetic risk factor, increasing the risk in three- to eightfold [4]. In addition, recent whole genome and whole exome analyses have identified rare coding variants in *TREM2* [5, 6], *PLD3* [7], *ABCA7* [8, 9], and *SORL1* [10, 11] that are associated with AD and confer risk comparable to that of carrying one *APOE* ε4 allele. Besides age at onset, the clinical presentations of LOAD and ADAD are remarkably similar with an amnestic and cognitive impairment phenotype [12, 13]. A minor fraction of cases of ADAD have additional neurological findings, sometimes also seen in LOAD [12, 13].

Altered cellular composition is associated with AD progression and decline in cognition. Neuronal loss in the hippocampus is characteristic in the initial stages of AD, which could explain early memory disturbances [14, 15]. As the disease progresses, neuronal death is observed throughout the cerebral cortex. Furthermore, ~ 25% of cognitively normal individuals who die by the age of ~ 75 years also presented substantial cerebral lesions that resemble AD pathology, including amyloid plaque, NFTs, and neuronal loss [16]. Thus, the identification of the brain cellular population structure is essential for understanding neurodegenerative disease progression [17]. However, stereology protocols for counting neurons can be tedious, require extensive training, and are susceptible to technical artifacts which may lead to biased quantification of cell-type distributions [17].

Recently there has been a growing interest in understanding the transcriptomic changes attributed to AD [18–25], as these may point to underlying molecular mechanisms of disease. These studies are typically designed to analyze the expression profiles of large cohorts ascertained from homogenized regions of the brain (e.g. bulk RNA-sequencing [RNA-seq]) of affected and control donors. However, as bulk RNA-seq captures the gene expression of all the constituent cells in the sampled tissue; the altered cellular composition associated with AD has been reported to confound downstream analyses [20].

Digital deconvolution approaches enhance the interrogation of expression profiles to identify the cellular population structure of individual samples, alleviating the requirement of additional neurostereology procedures. These approaches have been developed, tested, and applied to ascertain cellular composition altered in many traits [26–29]. However, digital deconvolution has not been applied to identify the cellular population structure from RNA-seq from human brain of AD cases and controls. Technical constraints restrict the dissociation of cells from the brains for very specific conditions [30–32]. Nevertheless, a limited number of RNA-seq from isolated cell populations from the brain have been generated [30–32]. Using these resources, we are now able to generate a reference panel for digital deconvolution of human brain bulk RNA-seq data.

We sought to investigate the cellular population structure in AD by analyzing RNA-seq from multiple brain regions of LOAD participants. To do so, we assembled a novel brain reference panel and evaluated the accuracy of digital deconvolution methods by analyzing additional cell-type-specific RNA-seq samples and by creating synthetic admixtures with defined cellular distributions. Then we analyzed large cohorts of pathologically confirmed AD cases and controls (n = 613) and verified that our model predicts cellular distribution patterns consistent with neurodegeneration. Finally, we generated RNA-seq from the parietal lobe of participants from the Charles F. and Joanne Knight Alzheimer’s Disease Research Center (Knight-ADRC) [33], including non-demented controls, LOAD cases, with enriched proportions of carriers of high-risk coding variants associated with AD, and also ADAD from The Dominantly Inherited Alzheimer Network [34] (DIAN). We compared the cell composition in ADAD and LOAD; and also evaluated differences among carriers of coding high-risk variants in *PLD3*, *TREM2*, and *APOE* ε4. Our findings indicate that cell-type composition differs among carriers of specific genetic risk factors, which might be revealing distinct pathogenic mechanisms contributing to disease etiology.

## Methods

### Subjects and samples

#### DIAN and Knight-ADRC

Parietal lobe tissue of post-mortem brain was obtained with informed consent for research use and was approved by the review board of Washington University in St. Louis. RNA was extracted from frozen brain using Tissue Lyser LT and RNeasy Mini Kit (Qiagen, Hilden, Germany). RNA-seq paired-end reads with read lengths of 2 × 150 bp were generated using Illumina HiSeq 4000 with a mean coverage of 80 million reads per sample (Table 1; Additional file 1: Table S1). RNA-seq was generated for 19 brains from DIAN, 84 brains with LOAD and 16 non-demented controls from Knight-ADRC [33]. The AD brains selected from Knight-ADRC are enriched for carrier of variants in *TREM2* (n = 20; Additional file 1: Table S1) and *PLD3* (n = 33; Additional file 1: Table S1). The clinical status of participants was neuropathologically confirmed [35]. We identified three additional participants from the Knight-ADRC study with PSEN1 (A79V, I143T, S170F) mutations. Clinical Dementia Rating (CDR) scores were obtained during regular visits throughout the study before the subject’s decease [36]. A range of other pathological measurements were collected during autopsy including Braak staging, as previously described [37].

**Table 1:** Demographics and disease status of cohorts from four brain bank resources

	Mayo	MSBB	DIAN	Knight-ADRC
Sample size	191	300	19	103
Age (years)	83 ± 7.77	83.3 ± 7.55	50.6 ± 7.06	85.1 ± 9.78
Male (%)	45.5	36	68.4	38.8
*APOE* ε4+ (%)	33.2	31.7	14.3	45.6
Brain weight	–	–	1187.7 ± 184.5	1138.1 ± 142.5
AD	82	135	19	87
PA	29	0	0	0
Control	80	85	0	16
CDR = 0	–	40	0	13
CDR = 0.5	–	40	0	9
CDR = 1	–	30	2	11
CDR = 2	–	44	4	14
CDR = 3	–	146	1	56

*Mayo* Mayo Clinic, *MSBB* Mount Sinai Brain Bank, *AD* Alzheimer’s disease, *PA* pathological aging, *CDR* Clinical Dementia Rating for available samples

RNA was extracted from frozen brain tissues using Tissue Lyser LT and RNeasy Mini Kit (Qiagen, Hilden, Germany) following the manufacturer’s instruction. RIN (RNA integrity) and DV200 were measured with RNA 6000 Pico Assay using Bioanalyzer 2100 (Agilent Technologies). The RIN is determined by the software on the Bioanalyzer taking into account the entire electrophoretic trace of the RNA including the presence or absence of degradation products. The DV200 value is defined as the percentage of nucleotides > 200 nt. RIN and DV200 for all the samples can be found on Additional file 1: Table S1. The yield of each sample is determined by the Quant-iT RNA Assay (Life Technologies) on the Qubit Fluorometer (Fisher Scientific). The complementary DNA (cDNA) library was prepared with the TruSeq Stranded Total RNA Sample Prep with Ribo-Zero Gold kit (Illumina) and then sequenced by HiSeq 4000 (Illumina) using 2 × 150 paired-end reads at McDonnell Genome Institute, Washington University in St. Louis with a mean of 58.14 ± 8.62 million reads. Number of reads and other quality control (QC) metrics can be found in Additional file 1: Table S1.

#### Mayo Clinic Brain Bank

Mayo Clinic Brain Bank RNA-seq was accessed from the Advanced Medicines Partnership – Alzheimer’s Disease (AMP-AD) portal (synapse ID = 5,550,404; accessed January 2017) (Table 1). Paired end reads of 2 × 101 base pairs were generated by Illumina HiSeq 2000 sequencers for an average of 134.9 million reads per sample. Neuropathology criteria, quality control procedures, RNA extraction, and sequencing details are explained elsewhere [18].

RNA-seq based transcriptome data were generated from post-mortem brain tissue collected from cerebellum (CB; 189 samples) and temporal cortex (TC; 191 samples) of Caucasian subjects [18, 38]. RNA was extracted using Trizol® reagent and cleaned with Qiagen RNeasy. RIN measurement was performed with Agilent Technologies 2100 Bioanalyzer. Samples with RIN > 5 were included. Library was prepared by Mayo Clinic Medical Genome Facility Gene Expression and Sequencing Cores with TruSeq RNA Sample Prep Kit (Illumina).

#### Mount Sinai Brain Bank

The Mount Sinai Brain Bank (MSBB) RNA-seq study was downloaded from the AMP-AD portal (synapse ID = 3,157,743; accessed January 2017) (Table 1). Single-end reads of 100 nt were generated by Illumina HiSeq 2500 System (Illumina, San Diego, CA, USA) for an average of 38.7 million reads per sample [39].

This dataset contains 1030 samples collected from four post-mortem brain regions of 300 subjects: anterior prefrontal cortex (APC; BA10); superior temporal gyrus (STG; BA22); parahippocampal gyrus (PHG; BA36); and inferior frontal gyrus (IFG; BA44). RNA-seq was generated using the TruSeq RNA Sample Preparation Kit v2 and Ribo-Zero rRNA removal kit (Illumina, San Diego, CA, USA) [39].

#### Induced pluripotent stem cell (iPSC)-derived neurons

Dermal fibroblasts were obtained from skin biopsies from research participants in the Knight-ADRC (Fibroblast lines: F11362, F12455, and F13504). Human fibroblasts were reprogrammed into iPSCs using non-integrating Sendai virus carrying OCT3/4, SOX2, KLF4, and cMYC [40, 41]. iPSCs were manually selected and expanded on Matrigel in mTesR1 (StemCell Techologies). iPSCs were characterized for expression of pluripotency markers by immunocytochemistry and quantitative polymerase chain reaction (qPCR). qPCR with probes specific to the Sendai virus were used to confirm the absence of virus in the isolated clones. All cell lines were confirmed to have a normal karyotype based on G-band karyotyping. To generate cortical neurons, iPSCs were plated in a v-bottom plate in neural induction media (StemCell Technologies; 65,000 per well) to form highly uniform neural aggregates. After five days, neural aggregates were transferred onto PLO/laminin-coated tissue culture plates. Neural rosettes formed over 5–7 days. The resulting neural rosettes were then isolated by enzymatic selection (StemCell Technologies) and cultured as neural progenitor cells (NPCs). NPCs were then differentiated by culturing in neural maturation medium (neurobasal medium supplemented with B27, GDNF, BDNF, cAMP). RNA was collected from the cells and sequenced following the same protocol and processing pipeline as the DIAN and Knight-ADRC dataset.

In addition, we accessed RNA-seq data generated for iPSC-derived neurons from the Broad iPSC study [42] (synapse ID: syn3607401). Forebrain neurons from wild-type background were generated using an embryoid body-based protocol to produce neural progenitor cells (day 17) and mature neurons (days 57 and 100). RNA was purified using a PureLink RNA mini-kit (Life Technologies). RNA-seq libraries were prepared using Illumina Strand Specific TruSeq protocol and sequenced to obtain an average of 75 M reads in paired reads per sample.

#### Translating ribosome affinity purification (TRAP)-seq mice

All animal procedures were performed in accordance with the guidelines of Washington University’s Institutional Animal Care and Use Committee. The Rosa26^fsTRAP^ mice (Gt(ROSA)26Sor^tm1(CAG-EGFP/Rpl10a,-birA)Wtp^) [43] (The Jackson Laboratory) were crossed with PV^Cre^ mice (Pvalb^tm1(cre)Arbr^) [44] (The Jackson Laboratory) to produce PV-TRAP mice directing expression of EGFP-L10a ribosomal fusion protein in parvalbumin (PV) expressing cells.

Purification of cell-type-specific messenger RNA (mRNA) by TRAP was described previously [45] with modifications. Briefly, PV-TRAP mouse brain was removed and quickly washed in ice-cold dissection buffer (1× HBSS, 2.5 mM HEPES-KOH (pH 7.3), 35 mM glucose, and 4 mM NaHCO_3_ in RNase-free water). Barrel cortex was rapidly dissected and flash-frozen in liquid nitrogen, and then stored at − 80 °C until use. Affinity matrix was prepared with 150 μL of Streptavidin MyOne T1 Dynabeads, 60 μg of Biotinylated Protein L, and 25 μg of each of GFP antibodies 19C8 and 19F7. The tissue was homogenized on ice in 1 mL of tissue-lysis buffer (20 mM HEPES KOH (pH 7.4), 150 mM KCl, 10 mM MgCl_2_, EDTA-free protease inhibitors, 0.5 mM DTT, 100 μg/mL cycloheximide, and 10 μL/mL rRNasin and Superasin). Homogenates were centrifuged for 10 min at 2000×*g*, 4 °C, and 1/9 sample volume of 10% NP-40 and 300 mM DHPC were added to the supernatant at final concentration of 1% (vol/vol). After incubation on ice for 5 min, the lysate was centrifuged for 10 min at 20,000×*g* to pellet insolubilized material. Then 200 μL of freshly resuspended affinity matrix was added to the supernatant and incubated at 4 °C for 16–18 h with gentle end-over-end mixing in a tube rotator. After incubation, the beads were collected with a magnet and resuspended in 1000 μL of high-salt buffer (20 mM HEPES KOH (pH 7.3), 350 mM KCl, 10 mM MgCl_2_, 1% NP-40, 0.5 mM DTT, and 100 μg/mL cycloheximide) and collected with magnets as above. After four times of washing with high-salt buffer, RNA was extracted using Absolutely RNA Nanoprep Kit (Agilent Technologies) following the manufacturer’s instructions. RNA quantification was measured using Qubit RNA HS Assay Kit (Life Technologies) and the integrity was determined by Bioanalyzer 2100 using an RNA Pico chip (Agilent Technologies). The cDNA library was prepared with Clontech SMARTer and then sequenced by HiSeq3000. Single-end reads of 50 base pairs were generated for an average of 29.2 million reads per sample (24 samples).

#### iPSC-derived microglia

The data were accessed from the AMP-AD portal (synapse ID: syn7203233). This dataset comprises iPSC-derived microglia (n = 10) from human primitive streak-like cells [46]. Within 30 days of differentiation, myeloid progenitors co-expressing CD14 and CX3CR1 were generated. These iPSC-derived microglia were able to perform phagocytosis and elicit ADP-induced intracellular Ca^2+^ transients that asserted their microglia identity as opposed to macrophage. Single-ended RNA-seq data were generated with the Illumina HiSeq 2500 platform following the Illumina protocol.

### RNA-seq QC and alignment

FastQC was applied to DIAN and Knight-ADRC RNA-seq data to perform quality checks on various aspects of sequencing quality [47]. The DIAN and Knight-ADRC dataset was aligned to human GRCh37 primary assembly using Star (ver 2.5.2b) [48]. We used the primary assembly and aligned reads to the assembled chromosomes, un-localized and unplaced scaffolds, and discarded alternative haploid sequences. Sequencing metrics, including coverage, distribution of reads in the genome [49], ribosomal and mitochondrial contents, and alignment quality, were further obtained by applying Picard CollectRnaSeqMetrics (ver 2.8.2) to detect sample deviation. Additional QC metrics can be found in Additional file 1: Table S1.

Aligned and sorted bam files were loaded into IGV [50] to perform visual inspection of target variants. Samples carrying unexpected variants or missing expected variants were labeled as potential swapped samples. In addition, variants were called from RNA-seq following BWA/GATK pipeline [51, 52]. The identity of the samples was later verified by performing IBD analysis against genomic typing from genome-wide association study chipsets.

### Expression quantification

We applied Salmon transcript expression quantification (ver 0.7.2) [53] to infer the gene expression for all samples included in the reference panel and participants in the Mayo, MSBB, DIAN, and Knight-ADRC. We quantified the coding transcripts of *Homo sapiens* included in the GENCODE reference genome (GRCh37.75). Similarly, we quantified the expression of the mice samples included in the reference panel using the *Mus musculus* reference genome (mm10).

### Reference panel

#### Reference samples

We assembled a cell-type-specific reference panel from publicly available RNA-seq datasets comprising both immunopanning collected or iPSC-derived neurons, astrocytes, oligodendrocytes, and microglial cells from human and murine samples. For immunopanning collected cells, antibodies for cell-type-specific antigens were utilized to bind and immobilize their targeted cell types in order to immunoprecipitate and purify each cell type from the suspensions [30]. cDNA synthesis was accomplished using Ovation RNA-seq system V2 (Nugen 7102) and library prepared with Next Ultra RNA-seq library prep kit from Illumina (NEB E7530) and NEBNext® multiplex oligos from Illumina (NEB E7335 E7500). TruSeq RNA Sample Prep Kit (Illumina) was used to prepare library for paired-end sequence on 100 ng of total RNA extracted from each sample. Illumina HiSeq 2000 Sequencer was used to sequence all libraries [30].

Both human adult TC tissue, collected from patients receiving neurological surgeries, and mice cells were disassociated, sorted and sequenced as described elsewhere [31], and deposited in the Gene Expression Omnibus GSE73721 and GSE52564. We also accessed neural progenitor cells (day 17) and mature human neurons (days 57 and 100) from Broad iPSC deposited in the AMP-AD portal [42] and neural progenitor cells and iPSC-derived neurons from [54]. Broad iPSC-derived neurons accessed from the AMP-AD portal were generated using an embryoid body-based protocol to differentiate into forebrain neurons. Wild-type cells used in the protocol were obtained from UConn StemCell Core. RNA was purified using PureLink RNA mini-kit (Life Technologies) and libraries were prepared by Broad Institute’s Genomics Platform using TruSeq protocol. Please refer to Additional file 1: Table S2 for additional information.

#### Marker genes

The reference panel was assembled with samples from four distinct cell types. A redundant set of well-known cell-type markers was selected from the literature [31, 55, 56] (Additional file 1: Table S3). Principal component analysis (PCA) was performed on the reference panel using R function *prcomp* (version 3.3.3) to verify that the expressions of these gene were clustering samples by their cell types (Additional file 1: Figure S1b**;** Additional file 1: Figure S2a).

### Inference of the cellular population structure

We ascertained alternative computation deconvolution algorithms implemented in the CellMix package (ver 1.6). Based on accuracy and robustness evaluation results, we compared and reported the following three algorithms that outperformed the others: Digital Sorting Algorithm (named “DSA”) [27], which employs linear modeling to infer cell distributions; the method population-specific expression analysis (PSEA, also named meanProfile in CellMix implementation) [29] that calculates estimated expression profiles relative to the average of the marker gene list for each cell type [29]; and a semi-supervised learning method that employs non-negative matrix factorization (ssNMF in CellMix implementation) [57]. We employed a leave-one-out cross-validation (LOOCV) procedure to evaluate the accuracy provided by each method. The best performing algorithm ssNMF integrates cell-type marker genes to resolve the drawbacks of completely unsupervised standard non-negative matrix factorization. We followed the standard procedure described in the CellMix package, which included the extraction of marker genes from the reference samples (function extractMarkers from the CellMix package), and the posterior invocation of the function *ged* to infer cellular population from the gene expression of bulk RNA-seq data. Besides, we tested additional methods which provided considerably lower accuracy (least-squares fit [58], quadratic programing [59]) or no significant difference (support vector regression [26] or latent variable analysis [60]) to the methods presented.

We selected the reference samples that provide the most faithful transcriptomic profile for their respective cell types by following a LOOCV approach. We trained iteratively deconvolution models using all but one of the samples that was tested. Only samples predicted with a composition > 80% were kept for the reference panel (Additional file 1: Table S2; Additional file 1: Figure S2b).

### Accuracy and robustness evaluation

#### Chimeric validation

To emulate heterogeneous tissue with known and controlled cellular composition, we generated chimeric libraries pooling reads (to a total of 400,000) contributed from the human reference samples (see Additional file 1: Table S2). This process was repeated 720 times, using alternative reference samples to model each cell type. The proportion of reads that the libraries of neurons, astrocytes, oligodendrocytes, and microglia provided to the chimeric libraries varied in predefined ranges (Additional file 1: Figure S3). As a result, each of the chimeric libraries contained reads that followed 32 different distributions (neuronal reads contributed 2–36% of reads, astrocytes 22–76%, oligodendrocytes 6–62%, and microglia 1–5%). Refer to Additional file 1: Table S4 for detailed description of the 32 different distributions. We quantified the chimeric reads using Salmon (v0.7.2) [53] and employed the reference samples that did not contribute reads to the chimeric library as reference panel for the deconvolution methods.

Overall, we quantified the expression of 23,040 (720 × 32) chimeric libraries. We evaluated the accuracy using the root-mean-square error (RMSE, Eq. 1 to compare the digital deconvolution cellular proportion estimates (method ssNMF) vs the defined proportion of reads specific to each of the chimeric libraries:

1 $$ RMSE=\sqrt{\frac{\sum_{i=1}^n{\left(\widehat{y}i- yi\right)}^2}{n}} $$

$$ \widehat{y}i- estimated\ value, yi- observed\ value $$

We also tested whether the deconvolution results were dominated by the expression of any specific marker gene and ascertained the robustness of the inferred cellular population structure to any possibly altered expression of marker genes. To do so, we performed the deconvolution analysis discarding each of the marker genes one at a time and evaluated how these distributions differed in comparison to the full gene reference panel.

### Statistical analysis

We employed linear regression models to test the association between cell-type proportions and disease status (R Foundation for Statistical Computing, ver.3.3.3). We used stepwise discriminant analysis (stepAIC function of R package MASS, version 7.3–45) to determine significant covariates and to correct for confounding effects. We included RIN, batch, age at death, and post-mortem interval (PMI) as covariates for the Mayo Clinic analyses. For MSBB analyses, we corrected for RIN, PMI, race, batch, and age at death. We also used linear-mixed models to perform multiple-region association analysis, employing random slopes and random intercepts grouping by observations and by donors [61], and correcting for the same covariates previously described.

To analyze the DIAN and Knight-ADRC studies, we applied linear-mixed models (function lmer and Anova, R packages lme4 ver.1.1 and car ver.2.1, respectively), clustering at family level to ascertain the effect of the neuropathological status in the cell proportion and corrected for RIN and PMI. For late-onset specific analyses we also corrected for age at death.

Cellular composition shown as proportions were plotted using R package ggplot2 (ver 2.2.1).

## Results

### Study design

**Fig. 1:**
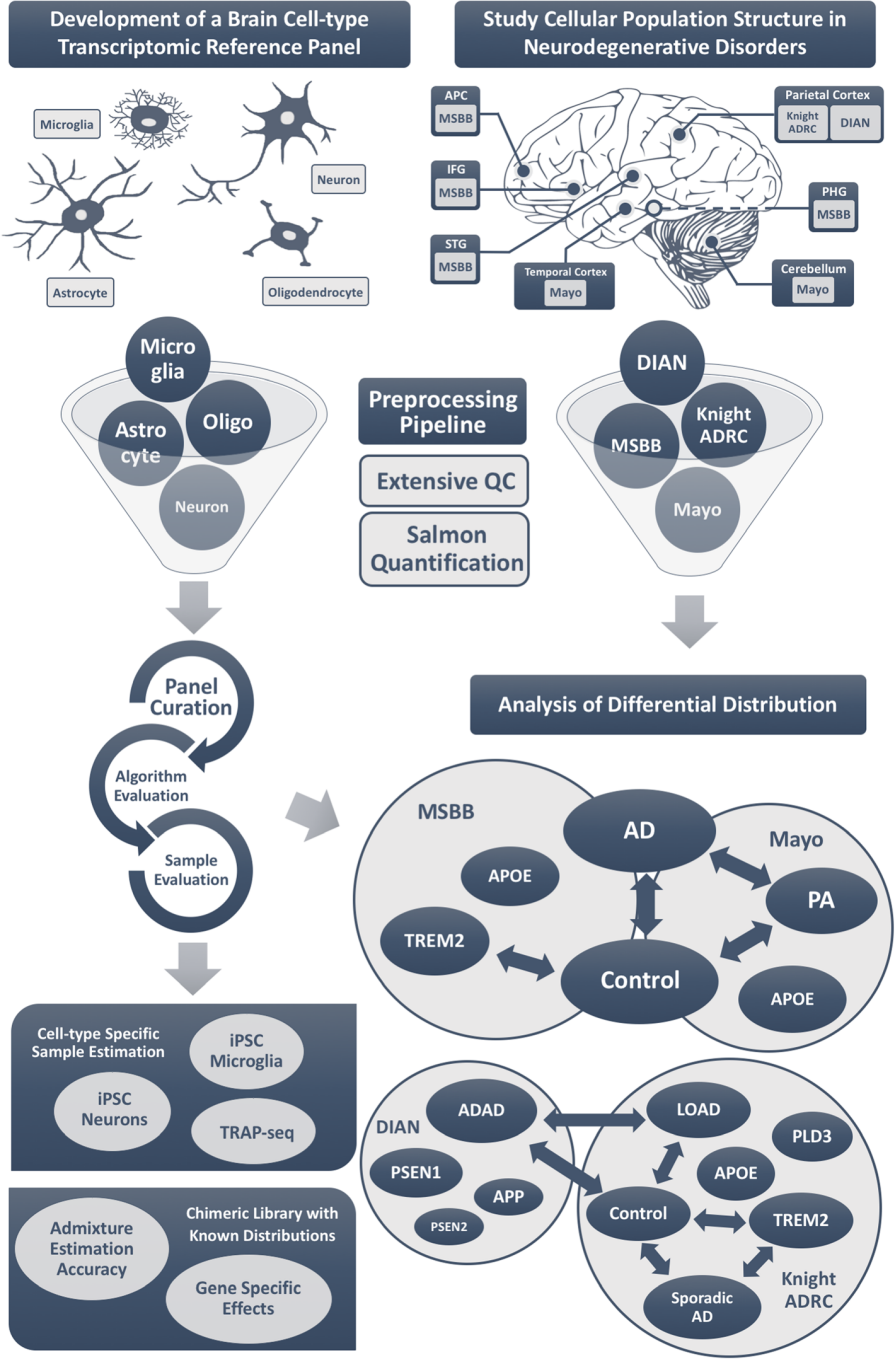
Study design development of the brain cell-type transcriptomic reference panel (*left column*): the expression signatures of key cell types of the brain were curated by compiling publicly available RNA-seq data from neurons, astrocytes, oligodendrocytes, and microglia. The panel was curated iteratively to retain only those samples that showed the most faithful expression signature, while evaluating alternative digital deconvolution methods. The accuracy of digital deconvolution to estimate brain cellular proportion was validated using additional cell-type-specific samples and also by generating chimeric libraries. To study cellular population structure in AD (*right column*), we accessed publicly available data from the AMP-AD, including Mayo Clinic and MSBB datasets. In addition, we generated RNA-seq from participants of the Knight-ADRC and DIAN studies. These three studies generated RNA-seq data from PA brains, AD cases, and neuropath-free controls in a total of six cerebral cortex regions and cerebellum. We quantified the gene expression for all of the samples included in these studies using the same RNA-seq processing pipeline. Using digital deconvolution methods, we estimated the brain cellular proportions of the samples and compared the proportion between AD cases and controls. We studied the cell structure of brain carriers of Mendelian pathological mutations and variants that confer high-risk to AD. APC anterior prefrontal cortex, STG superior temporal gyrus, PHG parahippocampal gyrus, IFG inferior frontal gyrus, MSBB Mount Sinai Brain Bank, AD Alzheimer’s disease, PA pathological aging

To infer cellular composition from RNA-seq, we first assembled a reference panel to model the transcriptomic signature of neurons, astrocytes, oligodendrocytes, and microglia. The panel was created by analyzing expression data from purified cell lines. We evaluated alternative digital deconvolution methods and selected the best performing for our primary analyses. We tested the digital deconvolution accuracy on iPSC-derived neurons/microglia cells and neuronal TRAP-seq (Fig. 1). Finally, we verified its accuracy by creating artificial admixture with pre-defined cellular proportions.

Once the deconvolution approach was optimized, we calculated the cell proportion in AD cases and controls from the different brain regions of Mayo and MSBB datasets. The RNA-seq data for the Mayo Clinic study (*n* = 191) [18] and MSBB (*n* = 300) [39] are deposited in the AMP-AD knowledge portal (synapse ID: syn5550404 and syn3157743; Table 1). The Mayo study includes RNA-seq from the TC and CB for AD affected and non-demented controls, in addition to pathological aging (PA) participants (Fig. 1). The MSBB also profiled four additional cerebral cortex areas: APC; STG; PGH; and IFG; Table 1 and Fig. 1). We restricted the case-control analysis to subjects with definite AD and autopsy-confirmed controls. In addition, we generated RNA-seq from parietal lobe for participants of the Knight-ADRC (84 late-onset cases, carriers of genetic risk factors and 16 controls; Additional file 1: Table S1) and The Dominantly Inherited Alzheimer Network (DIAN; 19 carriers of mutations in *APP*, *PSEN1*, *PSEN2*) (Table 1; Fig. 1)**.** We employed the same pipeline to process all of the samples in order to avoid any bias. Furthermore, RNA-seq from the Knight-ADRC and DIAN studies allowed us to compare the cell composition from ADAD vs LOAD brains, and similarly to test for differences in brains of controls, sporadic AD who do not carry any known high-risk variant, carriers of high-risk variants in *TREM2* (*n* = 20), *PLD3* (*n* = 33), and *APOE* ε4 allele.

### Development of a reference panel to estimate brain cellular population structure

Due to limited availability of brain cell-type-specific transcriptomic data, we compiled reference samples from different sources, including single-population RNA-seq from mice and human (immunopan-purified oligodendrocytes, neurons, astrocytes and microglia, and iPSC-derived neurons and astrocytes).

We first tried to create a transcriptome-wide reference panel by selecting the genes that are differentially expressed among cell types [26, 60, 62]. However, the species heterogeneity of the reference samples we compiled ruled out this attempt, as the PCA showed that differences between the human and mice donor samples dominated the transcriptome-wide profiles (Additional file 1: Figure S1a). For this reason, we curated a list of marker genes that have been described to tag these distinct cell types [31, 55, 56] (Additional file 1: Table S3). A visual inspection of the expression of these marker genes in the samples we compiled suggested a divergent transcriptomic profile among the cell types (Additional file 1: Figure S2a). The PCA showed that their expression was sufficient to cluster samples of neurons, astrocytes, oligodendrocytes, and microglia with their respective cell types, regardless of the species of the reference samples (Additional file 1: Figure S1b; Additional file 1: Table S2). We observed that some samples did not cluster with their expected cell types and coincidently the LOOCV indicated that these samples had an expression signatures that differed from the other samples of the same cell type. However, we found that all of these outliers correspond to samples not correctly purified or that were sequenced in early stages of differentiation (Additional file 1: Supplementary Results). After discarding these samples, we assessed six digital deconvolution algorithms implemented in the CellMix package [62] and found that the ssNMF [57] calculated the most accurate estimates (see “Methods”). Our final reference panel (Additional file 1: Table S2; Additional file 1: Table S3) had a very high confidence to predict cell types with a mean predicted accuracy = 95.2%, s.d. = 4.3 (Additional file 1: Figure S2b), and a RMSE = 0.06 (Additional file 1: Table S5).

### Optimization, validation, and accuracy estimation of the reference panel and digital deconvolution method

Once we identified the optimal approach to perform digital deconvolution from brain RNA-seq, we benchmarked it by using three sets of independent pure cell populations and simulated chimeric libraries.

We first validated the accuracy to predict neuronal composition by generating RNA-seq for eight iPSC-derived cortical neurons (see “Methods”). We observed an accurate prediction in these independent cell lines (mean neuronal proportion = 94.8%, s.d. = 1.1%; Additional file 1: Figure S4a). We also ascertained the cellular composition of mRNA extracted from the barrel cortex neurons isolated by TRAP in 24 mice. TRAP is a method that captures cell-type-specific mRNA translation by purifying tagged ribosomal subunit and capturing the mRNA it bound to [45]. We observed an average of neuronal proportion = 96.7% and s.d. = 1.2% (Additional file 1: Figure S4b). Similarly, we assessed the RNA-seq data generated for iPSC-derived microglia (n = 10) deposited in the AMP-AD portal (synapse ID: syn7203233) and inferred their cellular population structure and observed a mean microglia proportion = 86.6% and s.d. = 7.1% (Additional file 1: Figure S4c).

To evaluate the accuracy of digital deconvolution for measuring cell-type proportion from cell-type admixtures, we simulated RNA-seq libraries by pooling reads from individual cell types into well-defined proportions. We combined randomly sampled reads from neurons, astrocytes, oligodendrocytes, and microglia to create chimeric libraries that mimic bulk RNA-seq from brain, but with a range of pre-defined cell-type distributions (Additional file 1: Figure S3). We then quantified the gene expression for the chimeric libraries and inferred the cell-type distribution (employing for the reference panel samples that did not contribute reads to the chimeric libraries). This process was repeated 23,040 times, choosing distinct human samples to represent each cell type and varying the proportions in 32 alternative distributions (see “Methods” and Additional file 1: Table S4). The overall error (RMSE) compared to known proportions = 0.08.

Finally, we evaluated whether any gene included in the reference panel was dominating the inference of cell proportions. We re-calculated the cell-type distributions of the chimeric libraries but dropping each of the genes from the reference panel one at a time. We observed a negligible difference between the cellular population structure inferred using the full reference and the gene-dropped panels (average RMSE = 0.022, s.d. < 0.01). In this way, we verified that the proportions inferred using the reference panel are not driven by the expression of a single gene. This reassured us the inference should be robust to any bias introduced by the potential association of a single gene included in the reference panel with a particular trait.

### Deconvolution of bulk RNA-seq of non-demented and AD brains shows a characteristic signature for neurodegeneration

Pathologically, AD is associated with neuronal death and gliosis specifically in the cerebral cortex. We evaluated whether we could exploit deconvolution methods using our reference panel to detect altered cellular population structure from the bulk RNA-seq and whether this corresponded to known pathological alterations.

**Fig. 2:**
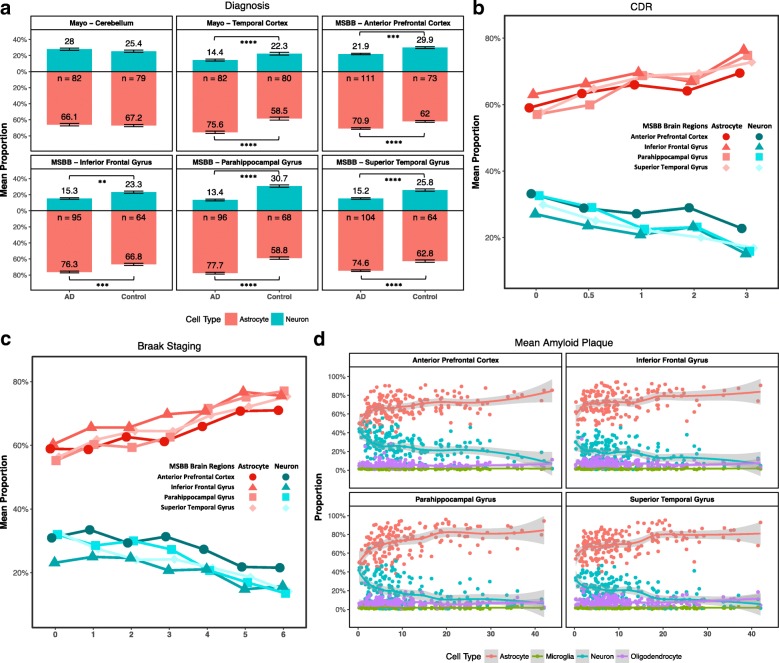
Cell-type distributions of the samples included in the Mayo Clinic and MSBB. Mean neuronal (*blue*) and astrocytic proportion (*red*) for **(a)** AD affected brains and controls (*bars* indicate standard deviations). The numbers of participants for each group are shown below the *x-axis*. Distribution for additional clinical and pathological phenotypes reported for the MSBB: **(b)** CDR scores and **(c)** Braak staging. **d** Brain cell-type proportions (*x-axis*) plotted against the mean number of amyloid plaque (values > 0; *y-axis*). Standard errors were depicted in *shaded area* with LOESS smooth curve fitted to cell-type proportions derived from deconvolution. (**p < 0.01; ***p < 1.0 × 10^−3^; and ****p < 1.0 × 10^−4^)

We initially analyzed the RNA-seq from the Mayo Clinic Brain Bank that includes bulk RNA-seq from the TC and CB for 191 participants [18] (Table 1). In the TC, we observed a significant higher astrocyte relative proportion (β = 0.23; *p* = 5.01 × 10^−09^; Table 2; Fig. 2; Additional file 1: Table S6) in AD brains compared to control brains. We also found a significant lower relative proportion of neurons (β = − 0.17; *p* = 1.58 × 10^−07^; Table 2; Fig. 2; Additional file 1: Table S6) and oligodendrocytes (β = − 0.07; *p* = 1.8 × 10^−02^; Table 2; Additional file 1: Figure S5; Additional file 1: Table S6). As expected given the absence of pathology, we did not observe a significant difference in the cell-type composition in the CB (Table 2).

**Table 2:** Comparison of the cellular population structure (AD vs neuropath-free controls) from the brains in the Mayo Clinic and Mount Sinai Brain Bank

	Brain regions	Sample size	Neuron		Astrocyte		Oligodendrocyte		Microglia	
		*n*	Effect	*p* value	Effect	*p* value	Effect	*p* value	Effect	*p* value
Mayo	AD vs Control									
	CB	119	− 0.03	2.74 × 10^−01^	0.05	8.65 × 10^−02^	− 0.02	1.07 × 10^−01^	−3.19 × 10^−04^	9.19 × 10^−01^
	TC	119	− 0.17	1.58 × 10^−07^	0.23	5.01 × 10^−09^	− 0.07	1.8 × 10^−02^	− 2.03 × 10^−03^	5.48 × 10^−01^
Mount Sinai Brain Bank	AD vs Control									
	APC	184	− 0.04	8.14 × 10^−04^	0.06	8.11 × 10^−05^	− 0.01	3.36 × 10^−02^	− 3.18 × 10^−03^	1.12 × 10^−02^
	STG	167	− 0.08	3.49 × 10^−07^	0.1	1.45 × 10^−07^	− 0.01	5.8 × 10^−02^	− 3.17 × 10^−03^	5.78 × 10^−02^
	PHG	160	− 0.11	1.35 × 10^−08^	0.13	5.48 × 10^−10^	− 0.02	1.79 × 10^−03^	− 3.18 × 10^−03^	1.35 × 10^−01^
	IFG	159	− 0.04	3.12 × 10^−03^	0.06	3.58 × 10^−04^	− 0.01	4.39 × 10^−02^	−3.98 × 10^−03^	1.64 × 10^−02^
	Clinical Dementia Rating									
	APC	184	− 0.02	9.38 × 10^−04^	0.02	2.07 × 10^−04^	− 3.43 × 10^−03^	1.25 × 10^−01^	− 1.46 × 10^−03^	4.95 × 10^−03^
	STG	167	− 0.03	1.87 × 10^−06^	0.04	3.33 × 10^−07^	− 0.01	2.1 × 10^−02^	−1.02 × 10^−03^	1.49 × 10^−01^
	PHG	160	− 0.04	8.56 × 10^−06^	0.04	2.85 × 10^−06^	− 0.01	8.7 × 10^−02^	− 1.94 × 10^−03^	2.53 × 10^−02^
	IFG	159	− 0.02	8.29 × 10^−05^	0.03	1.4 × 10^−05^	− 4.64 × 10^−03^	6.7 × 10^−02^	− 1.46 × 10^−03^	3.11 × 10^−02^
	Braak staging									
	APC	173	− 0.01	1.21 × 10^−02^	0.01	1.27 × 10^−03^	− 3.09 × 10^−03^	2.77 × 10^−02^	− 7.04 × 10^−04^	3.12 × 10^−02^
	STG	158	− 0.02	2.22 × 10^−07^	0.02	2.77 × 10^−07^	− 2.91 × 10^−03^	1.17 × 10^−01^	− 5.47 × 10^−04^	1.97 × 10^−01^
	PHG	147	− 0.02	1.83 × 10^−06^	0.03	9.6 × 10^−08^	− 0.01	1.49 × 10^−03^	− 3.71 × 10^−04^	4.97 × 10^−01^
	IFG	152	− 0.01	1.01 × 10^−02^	0.01	8.56 × 10^−04^	− 3.55 × 10^−03^	2.37 × 10^−02^	− 1.01 × 10^−03^	1.74 × 10^−02^
	Mean amyloid plaques									
	APC	184	−1.88 × 10^−03^	3.6 × 10^−03^	2.82 × 10^−03^	1.03 × 10^−04^	− 7.99 × 10^−04^	2.13 × 10^−03^	− 1.46 × 10^−04^	1.72 × 10^−02^
	STG	167	−4.2 × 10^−03^	7.73 × 10^−08^	0.01	4.63 × 10^−08^	− 6.08 × 10^−04^	9.01 × 10^−02^	− 2.04 × 10^−04^	1.5 × 10^−02^
	PHG	160	− 4.96 × 10^−03^	5.05 × 10^−09^	0.01	1.26 × 10^−10^	− 9.99 × 10^− 04^	1.85 × 10^−03^	− 2.1 × 10^−04^	2.58 × 10^−02^
	IFG	159	−2.58 × 10^−03^	3.82 × 10^−04^	3.53 × 10^−03^	1.96 × 10^−05^	− 7.41 × 10^−04^	1.51 × 10^−02^	− 2.04 × 10^−04^	1.26 × 10^−02^

The cell-type proportions from AD cases and control were inferred from bulk RNA-seq using the ssNMF method. Effects of AD and associations with additional clinical and pathological phenotypes in cell-type distributions were estimated using linear regression model

*CB* cerebellum, *TC* temporal cortex, *APC* anterior prefrontal cortex, *SGT* superior temporal gyrus, *PHG* parahippocampal gyrus, *IFG* inferior frontal gyrus

The distribution of microglia was similar in the TC and CB from AD and control brains (Table 2; Additional file 1: Figure S5). The proportion of microglia was lower than any other cell types. The Mayo dataset also includes brains from individuals with PA (Table 1); which is neuropathologically defined by amyloid-beta (Aβ) senile plaque deposits but little or no neurofibrillary tau pathology [18, 63]. We observed a significant lower relative proportion of microglia in PA brains compared to AD in both TC and CB (Additional file 1: Table S7; Additional file 1: Figure S6). Therefore, we speculated that the lack of changes in the AD microglial population was neither due to low statistical power nor the inability of our method to estimate the microglial proportions but reflected unaltered neuropathological observations in AD brains.

We also analyzed data from the MSBB, which contains bulk RNA-seq for four additional cerebral cortex areas (APC, STG, PHG, IFG). Replicating our findings from the Mayo dataset, we observed a significant lower relative proportion in neurons and increase in astrocytes in all four areas (Table 2; Fig. 2; and Additional file 1: Table S6). The strongest effect size was detected in the PHG and STG (*p* < 3.49 × 10^−07^) (Table 2; Additional file 1: Table S8). Neuropathological studies have described that the PHG is one of the first brain areas in which AD pathology occurs [64–66]. We also observed a significant and strong correlation between neuronal and astrocyte relative proportions and the last ascertained clinical status (CDR), the number of amyloid plaques and Braak staging (Table 2; Fig. 2; Additional file 1: Figure S7).

### The cellular population structure differs between ADAD vs LOAD

While the loss of neurons is a common feature of AD, it is not clear whether the mechanism holds true across different forms of AD or AD cases carrying different genetic risk variants. Therefore, we investigated whether AD with distinct etiologies showed different cellular compositions. We generated RNA-seq data from the parietal lobe of participants enrolled in Knight-ADRC (84 LOAD, 3 ADAD, and 16 neuropath-free controls) and DIAN (19 ADAD) studies (Table 1; Additional file 1: Table S1). We selected the LOAD and ADAD participants to match for CDR at death, brain weight, and sex distributions (see Additional file 1: Table S1).

**Fig. 3:**
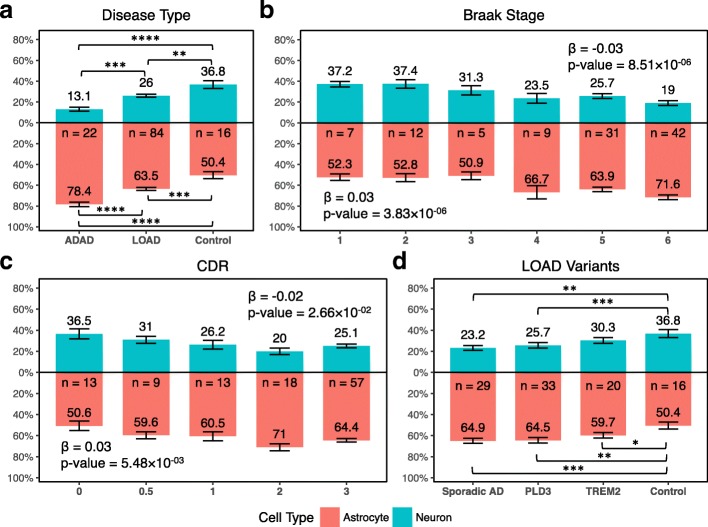
Neuron and astrocyte distributions from the DIAN and Knight-ADRC brains. **a** Mean neuronal (*blue*) and astrocytic (*red*) proportions for carriers of pathogenic mutations in *APP*, *PSEN1*, or *PSEN2* (ADAD), late-onset AD (LOAD), and neuropath-free controls (*bars* indicate standard deviations). Neuronal and astrocytic proportions plotted against **(b)** Braak staging and **(c)** by CDR. **d** Cell-type distributions for carriers of AD genetic risk factors. *Lines* indicate significance levels (*p < 0.05; **p < 0.01; ***p < 1.0 × 10^−3^; ****p < 1.0 × 10^−4^)

Using digital deconvolution, we determined the cellular composition for these brains. We observed a significant lower relative proportion of neurons (β = − 0.02, *p* = 2.66 × 10^−02^) and significant higher relative proportion of astrocyte in AD (β = 0.03, *p* = 5.48 × 10^−03^) for the combined LOAD and ADAD brains compared to controls (Table 3; Fig. 3; Additional file 1: Table S9), consistent with our findings in the Mayo and MSBB datasets. Similarly, the joint analysis of the brains from Knight-ADRC and DIAN showed significant associations between the neuronal and astrocyte relative proportions and neuropathological measures (Braak staging: β = − 0.03, *p* = 8.51 × 10^−06^ for neurons and β = 0.03, *p* = 3.83 × 10^−06^ for astrocytes; Table 3; Fig. 3b) as well as for clinical measures (CDR: β = − 0.02, *p* = 2.66 × 10^−02^ for neurons and β = 0.03 and *p* = 5.48 × 10^−03^ for astrocytes; Table 3; Fig. 3c). We did not observe a significant difference in the compositions of microglia or oligodendrocytes (Table 3; Additional file 1: Figure S8).

**Table 3:** Cellular population structure altered in the parietal lobe from AD brains in the DIAN study and Knight-ADRC brain bank

Disease status	Sample size	Neuron		Astrocyte		Oligodendrocyte		Microglia	
	*n*	Effect	*p* value	Effect	*p* value	Effect	*p* value	Effect	*p* value
AD status									
AD^a^ vs Control	122	− 0.11	5.52 × 10^−04^	0.14	2.48 × 10^−05^	− 0.03	6.5 × 10^−02^	− 2.64 × 10^−03^	2.49 × 10^−01^
ADAD vs Control	38	− 0.19	3.94 × 10^−07^	0.24	1.57 × 10^−10^	− 0.04	8.5 × 10^−03^	− 0.01	7.77 × 10^−05^
LOAD vs Control	100	− 0.09	5.67 × 10^−03^	0.12	3.34 × 10^−04^	− 0.02	1.06 × 10^−01^	− 1.70 × 10^−03^	4.57 × 10^−01^
ADAD vs LOAD									
Braak matched	42	− 0.08	1.03 × 10^−02^	0.11	9.26 × 10^−04^	− 0.03	7.1 × 10^−02^	− 1.46 × 10^−03^	7.01 × 10^−01^
Braak corrected	91	− 0.09	4.71 × 10^−03^	0.11	5.24 × 10^−04^	− 0.02	1.77 × 10^−01^	− 2.41 × 10^−03^	4.25 × 10^−01^
CDR corrected	94	− 0.12	2.11 × 10^−03^	0.13	6.29 × 10^−04^	− 0.02	3.8 × 10^−01^	− 3.11 × 10^−03^	2.41 × 10^−01^
Clinical Dementia Rating									
AD^a^ and Controls	110	− 0.02	2.66 × 10^−02^	0.03	5.48 × 10^−03^	− 0.01	2 × 10^−01^	− 4.63 × 10^−04^	4.77 × 10^−01^
ADAD and Controls	26	− 0.08	4.12 × 10^−04^	0.11	1.78 × 10^−07^	0.01	4.03 × 10^−03^	− 1.55 × 10^−03^	1.75 × 10^−08^
LOAD and Controls	100	− 0.02	3.22 × 10^−02^	0.03	7.01 × 10^−03^	− 0.01	1.81 × 10^−01^	− 4.64 × 10^−04^	5.11 × 10^−01^
Braak staging									
AD^a^ and Controls	106	− 0.03	8.51 × 10^−06^	0.03	3.83 × 10^−06^	− 4.24 × 10^−03^	2.04 × 10^−01^	− 2.52 × 10^−04^	6.81 × 10^−01^
ADAD and Controls	33	− 0.05	2.37 × 10^−05^	0.06	2.45 × 10^−05^	− 0.01	2.29 × 10^−01^	− 7.2 × 10^−04^	4.89 × 10^−01^
LOAD and Controls	88	− 0.03	7.41 × 10^−04^	0.03	4.63 × 10^−04^	− 3.72 × 10^−03^	3.29 × 10^−01^	− 1.66 × 10^−04^	7.86 × 10^−01^

^a^AD includes both autosomal dominant AD (ADAD) and late-onset AD (LOAD)

The cellular population structure was inferred using the ssNMF method. Effects and *p*-values for the association with disease status, clinical dementia rating and Braak staging using generalized mixed models. We identified similar trends with approximately the same significance levels

*AD* Alzheimer’s disease, *ADAD* autosomal dominant AD, *LOAD* late-onset AD

Next, we compared the cell proportion of LOAD vs ADAD and found that the cell composition differs between them. We first selected the LOAD brains (*n* = 25) to match the Braak staging distribution of ADAD brains (*n* = 17). The ADAD brains showed a significant lower relative neuronal proportion compared to LOAD brains (β = − 0.08; *p* = 1.03 × 10^−02^; Table 3) and an increased relative astrocyte proportion (β = 0.11; *p* = 9.26 × 10^−04^; Table 3). Then, we analyzed the entire Knight-ADRC LOAD brains, by extending the model to correct for Braak stages. We also observed significant lower relative neuronal proportion (β = − 0.09; *p* = 4.71 × 10^−03^; Table 3; Fig. 3a; Additional file 1: Table S9) and increased relative astrocyte proportion (β = 0.11; *p* = 5.24 × 10^−04^; Table 3; Fig. 3a; Additional file 1: Table S9) in ADAD brains compared to LOAD. We observed the same cellular differences when we corrected for CDR at death (β = − 0.12; *p* = 2.11 × 10^−03^ for neurons and β = 0.13; *p* = 6.29 × 10^−04^ for astrocytes; Table 3; Fig. 3b, c). In summary, our results indicate that ADAD individuals present a higher neuronal loss even in the same stage of the disease, suggesting that in ADAD neuronal death plays a more important role in pathogenesis compared to sporadic AD, in which other factors such as inflammation or immune response may be involved.

### Specific genetic variants confer a distinctive cell composition profile

A variety of genetic variants increase risk of LOAD; however, it is unclear if the cellular mechanisms are the same across these distinct risk factors. Therefore, we tested the hypothesis that distinct genetic causes of LOAD have characteristic cellular population signatures.

**Fig. 4:**
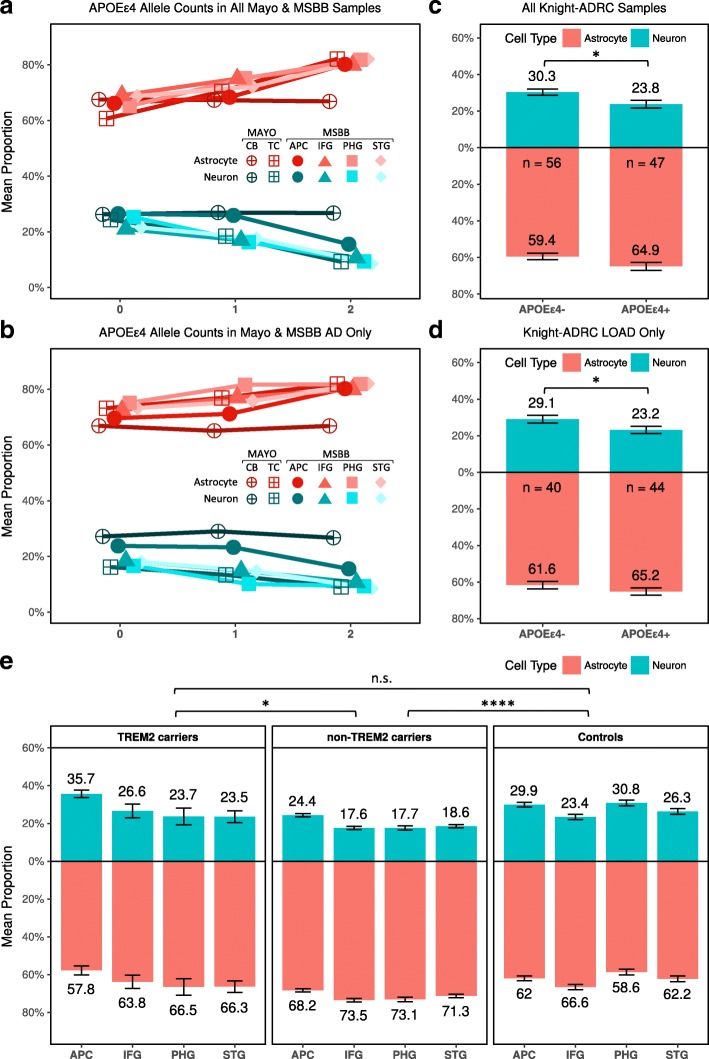
Effect of the *APOE* ε4 allele and *TREM2* coding variants on the cellular population structure. Mean neuronal (*blue*) and astrocytic (*red*) proportions for **(a)** AD cases and controls in the Knight-ADRC brains categorized by *APOE* ε4 carriers vs non-carriers and **(b)** AD cases of Knight-ADRC brain bank (*bars* indicate standard deviations). **c** AD cases and controls in the Mayo Clinic and MSBB. **d** AD cases in the Mayo Clinic and MSBB. **e** Neuronal (*blue*) and astrocyte (*red*) distributions for samples included in the MSBB stratified by *TREM2* genetic status. APC anterior prefrontal cortex, STG superior temporal gyrus, PHG parahippocampal gyrus, IFG inferior frontal gyrus (n.s. *p* > 0.05; *p < 0.05; ****p < 1.0 × 10^−4^)

We initially ascertained the effect of *APOE* ε4 on the cell-type composition. We observed a significant lower relative proportion of neurons (β = − 0.06 for each of the ε4 alleles; *p* = 9.91 × 10^−03^) and increase of relative proportion of astrocytes (β = 0.10; *p* = 4.15 × 10^−02^) from the TC included in the Mayo Clinic dataset (Additional file 1: Table S10; Fig. 4a; Additional file 1: Figure S9a). This finding was replicated when we performed a multi-area analysis of the MSBB dataset (β = − 0.04; *p* = 2.60 × 10^−03^ and β = 0.05; *p* = 1.31 × 10^−03^ for neurons and astrocytes, respectively; Table 4; Fig. 4a; Additional file 1: Table S10; Additional file 1: Figure S9a). Given the strong risk conferred by the *APOE* ε4 allele [4], we studied its effects on the cell-type composition by restricting our analysis to AD brains. We observed a significant association in the multi-area analysis of the MSBB dataset (β = − 0.03 *p* = 4.01 × 10^−02^; Table 4; Fig. 4b; Additional file 1: Table S11; Additional file 1: Figure S9b) and also a significant increase in relative proportion of astrocytes (β = 0.03; *p* = 1.23 × 10^−02^; Table 4; Fig. 4b; Additional file 1: Table S11; Additional file 1: Figure S9b). We also observed a significant decrease in relative proportion of neurons (β = − 0.06; *p* = 2.11 × 10^−02^; Table 4; Fig. 4c) when we analyzed the LOAD and control brains from the Knight-ADRC. When we restricted the analysis to AD brains from the Knight-ADRC and compared the *APOE* ε4 carriers (*n* = 44) to non-carriers (*n* = 40) we also observed a decreased relative neuronal proportion (β = − 0.06; *p* = 2.69 × 10^−02^; Table 4; Fig. 4d). We extended the models to correct for the Braak stages and observed a significant association for the relative proportion of neurons with the *APOE* ε4 allele in the Knight-ADRC dataset (β = − 0.06; *p* = 3.66 × 10^−02^; Table 4) and a significant association for the relative proportion of astrocytes in the MSBB (β = 0.04; *p* = 4.89 × 10^−02^; Table 4). Furthermore, we performed a meta-analysis to combine the evidence of both studies and observed a significant association of the relative neuronal proportion with *APOE* ε4 allele (*p* = 1.86 × 10^−02^) and marginally significant association for the relative astrocytic relative proportion (*p* = 0.09).

**Table 4:** Gene-specific cellular proportion analysis for Knight-ADRC and Mount Sinai Brain Bank studies

Variant carriers	Sample size	Neuron		Astrocyte		Oligodendrocyte		Microglia	
	*n*	Effect	*p* value	Effect	*p* value	Effect	*p* value	Effect	*p* value
Knight-ADRC									
*PLD3* vs Control	49	− 0.1	1.6 × 10^−04^	0.13	2.84 × 10^−03^	− 0.03	6.17 × 10^−02^	7.05 × 10^−04^	7.89 × 10^− 01^
*TREM2* vs Control	36	− 0.07	7.93 × 10^−02^	0.11	1.05 × 10^−02^	− 0.03	4.9 × 10^−02^	1.65 × 10^−03^	5.84 × 10^− 01^
Sporadic AD vs Control	45	− 0.11	5.45 × 10^−03^	0.13	2.95 × 10^−04^	− 0.02	4.55 × 10^−01^	− 3.48 × 10^−03^	1.13 × 10^−01^
*APOE*ε4+ vs *APOE*ε4- LOAD cases & controls	100	− 0.06	2.11 × 10^−02^	0.05	5.35 × 10^−02^	0.01	3.72 × 10^−01^	− 8.09 × 10^−04^	6.31 × 10^−01^
*APOE*ε4+ vs *APOE*ε4- LOAD cases only	84	− 0.06	2.69 × 10^−02^	0.03	2 × 10^−01^	0.03	1.4 × 10^−02^	− 8.31 × 10^−04^	6.21 × 10^−01^
CDR corrected	84	− 0.06	2.78 × 10^−02^	0.03	2.05 × 10^−01^	0.03	1.16 × 10^−02^	−1.05 × 10^−03^	5.37 × 10^−01^
Braak corrected	73	− 0.06	3.66 × 10^−02^	0.03	3.72 × 10^−01^	0.03	4.51 × 10^−03^	− 1.14 × 10^−03^	5.93 × 10^−01^
Mount Sinai Brain Bank - Multi-region									
AD *TREM2* carriers vs Control	301	− 0.03	3.57 × 10^−01^	0.03	3.19 × 10^−01^	− 2.08 × 10^−03^	7.87 × 10^−01^	− 2.68 × 10^−03^	8.67 × 10^−02^
AD non-carriers *TREM2* vs Control	882	− 0.07	1.91 × 10^−08^	0.08	1.25 × 10^−08^	− 3.36 × 10^−03^	4.79 × 10^−01^	− 2.89 × 10^−04^	7.97 × 10^−01^
AD *TREM2* vs AD non-*TREM2*	673	0.05	1.98 × 10^−02^	− 0.05	1.58 × 10^−02^	2.12 × 10^−03^	7.76 × 10^−01^	− 2.13 × 10^−03^	1.74 × 10^−01^
CDR corrected	673	0.04	5.83 × 10^−02^	− 0.04	4.46 × 10^−02^	1.68 × 10^−03^	8.19 × 10^−01^	− 1.92 × 10^−03^	2.22 × 10^−01^
Braak corrected	642	0.05	1.3 × 10^−02^	− 0.05	2.7 × 10^−02^	− 1.82 × 10^−03^	8.13 × 10^−01^	− 2.66 × 10^−03^	1.28 × 10^−01^
Mean plaque counts corrected	673	0.05	2 × 10^−02^	− 0.05	1.59 × 10^−02^	1.73 × 10^−03^	8.15 × 10^−01^	− 2.2 × 10^−03^	1.5 × 10^−01^
*APOE*ε4 counts all samples	556	− 0.04	2.6 × 10^−03^	0.05	1.31 × 10^−03^	− 0.01	4.47 × 10^−02^	−3.58 × 10^−04^	6.53 × 10^−01^
*APOE*ε4 counts AD cases	225	− 0.03	4.01 × 10^−02^	0.03	4.23 × 10^−02^	− 4.52 × 10^−03^	3.73 × 10^−01^	− 5.13 × 10^−04^	6.78 × 10^−01^
CDR corrected	225	− 0.03	2.02 × 10^−02^	0.03	2.03 × 10^−02^	− 4.86 × 10^−03^	3.19 × 10^−01^	− 4.91 × 10^−04^	6.93 × 10^−01^
Braak corrected	198	− 0.03	7.35 × 10^−02^	0.04	4.89 × 10^−02^	− 0.01	8.54 × 10^−02^	−1.08 × 10^−03^	4.12 × 10^−01^

*AD* Alzheimer’s disease, *ADAD* autosomal dominant AD, *LOAD* late-onset AD, *CDR* Clinical Dementia Rating

Next, we analyzed the cellular composition in *PLD3* carriers (*n* = 33). *PLD3* carriers exhibited significantly lower relative proportion of neurons compared to controls (β = − 0.10; *p* = 1.60 × 10^−04^; Fig. 3d) and a significant higher relative proportion of astrocytes (β = 0.13; *p* = 2.84 × 10^−03^; Table 4; Fig. 3d). Sporadic AD non-carrier cases also exhibited significantly lower relative proportion of neurons compared to controls (β = − 0.11; *p* = 5.45 × 10^−03^) and significant higher relative proportion of astrocytes (β = 0.13; *p* = 2.95 × 10^−04^; Table 4; Fig. 3d). The cell proportion between sporadic AD non-carriers and *PLD3* carriers did not show any significant difference (*p* > 0.05).

Finally, we performed similar analyses with *TREM2* carriers. *TREM2* is involved in the immune response and its role in amyloid-β deposition or clearance remains controversial [67]. Our analysis on the Knight-ADRC data showed significantly higher relative astrocytic proportion in AD affected *TREM2* carriers (n = 20) compared to controls (β = 0.11; *p* = 1.05 × 10^−02^; Table 4; Fig. 3d). Despite *TREM2* carriers presenting lower neuron relative proportion compared to controls, this difference was not statistically significant (*p* > 0.05; Table 4; Fig. 3d). We analyzed whether the *TREM2* carriers provided sufficient power to detect a significant association. Our empirical estimates showed that *TREM2* sample size provides 96% of power to detect an association with an effect size comparable to that observed for sporadic AD (β = − 0.11). We also investigated the cellular proportion of the 11 *TREM2* carriers in the MSBB dataset. The multi-region analysis showed *TREM2* carriers do not show a significant difference in relative neuronal proportion compared to controls (*p* > 0.05; Table 4; Fig. 4e), whereas in the AD *TREM2* non-carriers the relative neuronal and astrocytic proportions are significantly different from controls (β = − 0.07; *p* = 1.91 × 10^−08^; and β = 0.08; *p* = 1.25 × 10^−08^ respectively; Table 4; Fig. 4e).

In fact, our analyses indicate that *TREM2* carriers have a unique cellular brain composition distinct than the other AD cases. *TREM2* brains showed significantly higher relative neuronal proportion (β = 0.05; *p* = 1.98 × 10^−02^) and significantly lower relative astrocyte proportion than the AD *non-*carries (β = − 0.05; *p* = 1.58 × 10^−02^; Table 4). The distribution of CDR, mean number of amyloid plaques, and Braak staging do not differ between strata. Nonetheless, we verified that the cellular proportions were still significantly different after correcting for each of those variables (Table 4). These results suggested that the mechanism that lead to disease in *TREM2* carriers is less neuron-centric than in the general AD population.

## Discussion

We have developed, optimized, and validated a digital deconvolution approach to infer cell composition from bulk brain gene expression that integrates publicly available cell-type specific expression data while addressing the heterogeneity of the phenotypic differences of samples and technical characteristics of transcriptome ascertainment. We acknowledge that the accuracy of this platform might be affected by the phenotypic diversity of the reference panel or the disease-induced dysregulation of genes it includes. However, the deconvolution approach proved to be robust to the genes included in the reference panel, as we demonstrated that the proportions it inferred are not driven by the expression of any single gene. This platform produced reliable cell proportion estimates, as was shown by the evaluation of independent datasets of iPSC-derived neurons and microglia, mice cortical neurons (Additional file 1: Figure S4), and simulated chimeric libraries.

We used this approach to deconvolve studies that include large numbers of neuropathologically defined AD and control brains with their transcriptome ascertained in distinct brain regions. We observed consistently significant lower relative neuronal proportion and increased relative astrocyte proportions in the cerebral cortex suggesting neuronal loss and astrocytosis. Compatible with other studies, we also identified that the altered cellular proportion is also significantly associated with decline in cognition and Braak staging [68]. In contrast, we did not identify a significant difference in the cellular population structure in the cerebellum, a region not affected in AD (Table 2; Fig. 2a).

We generated RNA-seq data from brains carrying pathogenic mutations in *APP*, *PSEN1*, and *PSEN2*, which cause alterations in Aβ processing and lead to ADAD, and also generated RNA-seq from brains of LOAD and neuropath-free controls. We observed altered cell composition in both ADAD and LOAD compared to controls. However, we identified that ADAD brains have a different cell-type composition than disease-stage-matched LOAD, as the ADAD has a significantly lower relative neuronal proportion and more pronounced astrocytosis. Given the specific cellular population structure of the *TREM2* carriers, we compared the neuronal and astrocytic relative proportion of ADAD to that of LOAD non-carriers of variants in *TREM2* and observed significant differences (β = − 0.09 and *p* = 6.89 × 10^−03^ for neurons and β = 0.10; *p* = 1.49 × 10^–03^ for astrocytes). This indicates that the difference of the relative proportion between ADAD and LOAD are not driven by *TREM2* carrier brains. Based on our results, we would hypothesize that this change in Aβ processing of ADAD would lead to more direct to neuronal death than the pathological processes of LOAD. Similarly, decreased neuronal and increased astrocyte relative proportions were significantly associated with *APOE ε4* allele. It has been reported *APOE* ε4 allele increases the risk for AD by affecting APP metabolism or Aβ clearance [69, 70], suggesting a direct link between APP metabolism and neuronal death.

In contrast, the analysis of the Knight-ADRC brains showed that the neuronal relative proportion decrease is less pronounced in *TREM2* carriers than in other LOAD cases. We replicated this finding in a multi-area analysis from the MSBB dataset. These results may implicate that *TREM2* risk variants lead to a cascade of pathological events that differ from those occurring in sporadic AD cases, which is also consistent with the known biology of *TREM2*. Further longitudinal neuroimaging analysis is required to validate our findings. *TREM2* is involved in AD pathology through microglia mediated pathways, implicated on altered immune response and inflammation [71]. Recent studies in *TREM2* knock-out animals showed that fewer microglia cells were found surrounding Aβ plaques with impaired microgliosis [72]. Furthermore, *TREM2* deficiency was reported to attenuate tauopathy against brain atrophy [73]. We found no significant difference in the proportion of microglia between AD cases and controls. However, we found significantly decreased microglia in brains exhibiting PA (Additional file 1: Table S7; Additional file 1: Figure S6), proving that these studies are sufficiently powered to identify significant differences. In any case, we cannot rule out the possibility of a change in the activation stage of microglia in these individuals. Overall, these results suggest that *TREM2* affects AD risk through a slightly different mechanism to that of ADAD or LOAD in general. Therefore, other pathogenic mechanisms should contribute to disease. We believe that a detailed modeling of immune response cells, reflecting the alternative microglia activation states, will generate more accurate profiles to elucidate the immune cell distribution in AD.

## Conclusions

There is a large interest in the scientific community to use brain expression studies to try to identity novel pathogenic mechanisms in AD and to identify novel therapeutic targets. These efforts are generating a large amount of bulk RNA-seq data, as single-cell RNA (scRNA-seq) from human brain tissue in large sample sizes is not feasible. Single-cell sorting needs to be performed with fresh tissue [74], which restrains the analysis of highly characterized fresh-frozen brains collected by AD research centers. Our results indicate that digital deconvolution methods can accurately infer relative cell distributions from brain bulk RNA-seq data, but we recognize the importance of obtaining traditional neuropathological measures to validate the results we observed. Having this approach validated for AD can have an important impact in the community, because digital deconvolution analyses can: (1) reveal distinct cellular composition patterns underlying different disease etiologies; (2) provide additional insights about the overall pathologic mechanisms underlying different mutations carriers for variants as in genes such as *TREM2*, *APOE*, *APP*, *PSEN1*, and *PSEN2*; (3) correct the effect that altered cell composition and genetic statuses have in addition to downstream transcriptomic analyses and lead to novel and informative results; and (4) help the analysis of highly informative frozen brains collected over the years.

In conclusion, our study provides a reliable approach to enhance our understanding of the fundamental cellular mechanisms involved in AD and enable the analysis of large bulk RNA-seq data that may lead to novel discoveries and insights into neurodegeneration.

## Additional file

Additional file 1: Supplementary results, tables and figures. (DOCX 2353 kb)

## Abbreviations

AD: Alzheimer’s disease
ADAD: Autosomal dominant Alzheimer’s disease
AMP-AD: Advanced Medicines Partnership - Alzheimer’s disease
APC: Anterior prefrontal cortex
Aβ: Amyloid-beta
CB: Cerebellum
CDR: Clinical Dementia Rating
DIAN: Dominantly Inherited Alzheimer Network
DSA: Digital sorting algorithm
IFG: Inferior frontal gyrus
iPSC: Induced pluripotent stem cell
Knight-ADRC: Charles F. and Joanne Knight Alzheimer’s Disease Research Center (Knight ADRC)
LOAD: Late-onset Alzheimer’s disease
meanProfile: Implementation of method Population-Specific Expression Analysis
MSBB: Mount Sinai Brain Bank
PA: Pathological aging
PCA: Principal component analyses
PHG: Parahippocampal gyrus
PSEA: Population-Specific Expression Analysis
RIN: RNA integrity number
RMSE: Root-mean-square error
ssNMF: Semi-supervised non-negative matrix factorization
STG: Superior temporal gyrus
TC: Temporal cortex
TRAP-seq: Translating ribosome affinity purification sequencing
