# Supplementary material for: Genetic variants associated with Alzheimer’s disease confer different cerebral cortex cell-type population structure
Source: Genome Med. 2018 Jun 8;10:43. doi: 10.1186/s13073-018-0551-4 (PMC5992755; doi:10.1186/s13073-018-0551-4)
Supplement: Supplementary file 1 — Supplementary results, tables and figures. (DOCX 2353 kb) [file 13073_2018_551_MOESM1_ESM.docx]

**Supplementary Results, Tables and Figures**

**Genetic variants associated with Alzheimer’s disease confer different cerebral cortex cell-type population structure**

Zeran Li^1^, Jorge L Del-Aguila^1^, Umber Dube^1,2^, John Budde^1^, Rita Martinez^1^, Kathleen Black^1^, Qingli Xiao^3^, Nigel J. Cairns^3,4,5^, The Dominantly Inherited Alzheimer Network (DIAN), Joseph D. Dougherty^1,7^, Jin-Moo Lee^3^, John C Morris^3,5,6^, Randall J. Bateman^3,5,6^, Celeste M. Karch^1^, Carlos Cruchaga^1,5,6,∏^ and Oscar Harari^1,∏^

Affiliations:

1.Department of Psychiatry, Washington University School of Medicine, 660 S. Euclid Ave. B8134, St. Louis, MO 63110, USA.

2. Medical Scientist Training Program, Washington University School of Medicine, 660 S. Euclid Ave., St. Louis, MO 63110, USA.

3.Department of Neurology, Washington University School of Medicine, 660 S. Euclid Ave., St. Louis, MO 63110, USA.

4. Department of Pathology & Immunology, Washington University in St. Louis, School of Medicine, 510 S. Kingshighway, MC 8131, Saint Louis, MO 63110, USA.

5.Knight Alzheimer’s Disease Research Center, Washington University School of Medicine, 660 S. Euclid Ave., St. Louis, MO 63110, USA.

6.Hope Center for Neurological Disorders. Washington University School of Medicine, 660 S. Euclid Ave. B8111, St. Louis, MO 63110, USA.

7.Department of Genetics, Washington University School of Medicine, 660 S. Euclid Ave., St. Louis, MO 63110, USA.

^∏^ To whom correspondence should be addressed.

**Supplementary Results**

**Deconvolution algorithm evaluation and leave-one-out validation**

To evaluate the performance of reference panel performance and test out different deconvolution algorithms, we employed and compared six digital deconvolution methods implemented in the CellMix package (ver 1.6) to infer cellular composition from reference samples RNA-seq data, including qprog [1], cs-qprog, DSA [2], ssFrobenius [3], meanProfile [4], deconf [5]. The deconvolution performance of reference panel was evaluated by following a leave-one-out cross-validation procedure to compare the predicted cellular composition with its expected cellular identity of each cell-type specific sample. The accuracy of this comparison was quantified using the root-mean-squared error (RMSE) calculation. A semi-supervised method adapted from non-negative matrix factorization [3] (ssNMF – named ssFrobenius in CellMix) generated the most accurate predictions; and we verified that similar results were obtained by the method population-specific expression analysis [4] (PSEA – named meanProfile in CellMix).

**Reference sample evaluation**

We selected 17 well accepted genes that tag brain cell types based on literature reviews [6-8] . The principal component analysis (**Fig S1b**) indicated that these genes can effectively cluster samples by their cell-type. We observed that first principal component (PC) captured the expression profile of astrocytes; as shown by the significant association of the expression of astrocyte marker genes with (p<8.05×10^-03^). The second PC captured the expression of genes whose expression is characteristic to oligodendrocytes (p<2.52×10^-02^). The third PC was negatively associated with neuronal genes (p<1.11×10^-05^) and positively with microglia (p<1.42×10^-02^).

Given the technical and biological heterogeneity of the samples we compiled for reference panel, we carried out an optimization phase to identify those samples that showed the most faithful expression profile to represent their respective cell types (See **Methods**). From the leave-one-out cross-validation results, we noticed that not all of the cell-type specific samples were predicted as expected (defined with a correct prediction proportion higher than 80%). Samples failed this criteria were due to various reasons, for example, the expression profile of immunopan-purified astrocytes collected from mice [8], human fetal [8] or human sclerotic hippocampal [8] brains were reported with altered expression [8, 9] that differed to an extent that could not be accurately ascertained by deconvolution methods. Similarly, neuronal proportion inferred from iPSC-derived neurons from schizophrenic donors [9] and iPSC-derived neurons collected at early stages of differentiation (< 100 days; Synapse ID: syn3607401) were also lower than 80%.

**Deconvolution is robust in down-sampled library compared to full-size library**

We ascertained the effect that sequencing depth has in the accuracy of deconvolution. We generated low-coverage versions (Picard DownsampleSam ver 2.8.2) of the samples that included a reduced number of randomly sampled reads (400,000 reads per sample), quantified the gene expression, inferred their cellular population proportions, and compared the distribution estimates with their full-depth libraries sequencing (more than 30 million reads per sample). We observed that the deconvolution was robust to the sequencing coverage, as shown by a correlation r^2^=0.98 (p < 2.2×10^-16^; **Fig S10**).

| **Table S1. Demographics and AD mutation carriers of DIAN and Knight-ADRC cohorts.** | | | | | |
| --- | --- | --- | --- | --- | --- |
|  | **DIAN & Knight-ADRC** | | | **Total DIAN &**  **Knight-ADRC** | **ADAD vs LOAD**  **t-tests p-value** |
|  | **ADAD** | **LOAD** | **Control** |  |  |
| **RIN** | 5.69 ± 1.13 | 6.44 ± 1.16 | 6.71 ± 1.18 | 6.34 ± 1.19 | 9.04×10^-03^ |
| **DV200** | 86.59 ± 4.12 | 89.48 ± 3.85 | 91.19 ± 2.54 | 89.18 ± 3.97 | 5.82×10^-03^ |
| **PMI** | 14.57 ± 10.29 | 13.05 ± 6.66 | 10.52 ± 6.09 | 12.99 ± 7.4 | 5.16×10^-01^ |
| **Age** | 51.27 ± 11.13 | 85.72 ± 6.83 | 87.08 ± 10.2 | 79.69 ± 15.67 | 2.61×10^-13^ |
| **Male %** | 0.64 | 0.39 | 0.38 | 0.43 | 4.68×10^-02^ |
| **APOE4+ %** | 0.3 | 0.52 | 0.06 | 0.44 | 1.94×10^-01^ |
| **CDR** | 2.2 ± 0.79 | 2.37 ± 0.93 | 0.22 ± 0.31 | 2.04 ± 1.14 | 5.42×10^-01^ |
| **Braak** | 5.94 ± 0.24 | 4.84 ± 1.29 | 1.93 ± 0.88 | 4.61 ± 1.62 | 8.81×10^-10^ |
| **Number of Total Reads (Million)** | 60.92 ± 5.6 | 57.7 ± 9.28 | 56.6 ± 7.98 | 58.14 ± 8.62 | 4.47×10^-02^ |
| **Uniquely Mapped Reads %** | 79.72 ± 4.28 | 80.74 ± 4.49 | 81.06 ± 5.96 | 80.6 ± 4.65 | 3.32×10^-01^ |
| **Mapped to Multiple Loci Reads %** | 16.39 ± 2.1 | 15.56 ± 2.2 | 15.08 ± 3.3 | 15.64 ± 2.36 | 1.07×10^-01^ |
| **Disease Status** | 22 | 84 | 16 | 122 | - |
| ***APP*** | 3 | 0 | 0 | 3 | - |
| ***PSEN1*** | 18 | 0 | 0 | 18 | - |
| ***PSEN2*** | 1 | 0 | 0 | 1 | - |
| ***TREM2*** | 0 | 20 | 0 | 20 | - |
| ***PLD3*^a^** | 0 | 33 | 0 | 33 | - |
| ***UNC5C*^a^** | 0 | 4 | 0 | 4 | - |
| **Sporadic AD** | 0 | 29 | 0 | 29 | - |
| **^a^** There are two Knight-ADRC subjects that carry both *PLD3* and *UNC5C* variants. | | | | | |

| **Table S2. Reference samples for each cell type.** GEO accession numbers for cell-type specific samples. | | | | |
| --- | --- | --- | --- | --- |
|  | **Reference Sample** | | | |
| **Type** | **Human** | | **Mouse** | **Human iPSC^a^** |
| **Neuron** | GSM1901333 | | GSM1269905 | YZ2-100day |
|  |  |  | GSM1269906 | YZ3-100day |
|  |  |  |  | YZ4-100day |
|  |  |  |  | YZ5-100day |
| **Astrocyte** | GSM1901309 | GSM1901317 | GSM1269903 | Astrocyte1 |
|  | GSM1901310 | GSM1901318 | GSM1269904 | Astrocyte2 |
|  | GSM1901311 | GSM1901319 |  |  |
|  | GSM1901312 | GSM1901320 |  |  |
|  | GSM1901313 | GSM1901321 |  |  |
|  | GSM1901314 | GSM1901322 |  |  |
|  | GSM1901315 | GSM1901323 |  |  |
|  | GSM1901316 | GSM1901324 |  |  |
| **Oligodendrocyte** | GSM1901335 | | GSM1269911 |  |
|  | GSM1901336 | | GSM1269912 |  |
|  | GSM1901338 | |  |  |
| **Microglia** | GSM1901339 | | GSM1269913 |  |
|  | GSM1901340 | | GSM1269914 |  |
|  | GSM1901341 | |  |  |
| **^a^** Samples accessed from the Broad iPSC cell-lines deposited in the AMP-AD [10]. | | | | |

| **Table S3. Gene markers for** **principal brain cell types**. | | |
| --- | --- | --- |
|  | **Cell Marker** | |
| **Type** | **Human** | **Mouse^a^** |
| **Neuron** | STMN2 | Stmn2 |
|  | SYN1 | Syn1 |
|  | SYT1 | Syt1 |
|  | GAD1 | Gad1 |
|  | CCK | Cck |
| **Astrocyte** | GFAP | Gfap |
|  | ALDH1L1 | Aldh1l1 |
|  | AQP4 | Aqp4 |
|  | GJA1 | Gja1 |
|  | SOX9 | Sox9 |
| **Oligodendrocyte** | MOG | Mog |
|  | MOBP | Mobp |
|  | SOX10 | Sox10 |
|  | GPR37 | Gpr37 |
| **Microglia** | TLR2 | Tlr2 |
|  | CX3CR1 | Cx3cr1 |
|  | IL1A | Il1a |
| **^a^** Mouse homologous genes were identified from Mouse Genome Database [11]. | | |

| **Table S4. Simulated chimeric tissue cell-type composition.** Percentages of reads contributed to the synthetic chimeric libraries. | | | | | | |  |
| --- | --- | --- | --- | --- | --- | --- | --- |
|  | | | **Percentage of reads** | | | |  |
| **Configurations** | **Neuron** | **Neuron** | | **Astrocyte** | **Oligodendrocyte** | **Microglia** | |
|  |  | **2.31** | | 51.7 | 43.5 | 2.49 | |
|  |  | **10** | | 72 | 15.2 | 2.81 | |
|  |  | **10.1** | | 44.7 | 42.9 | 2.31 | |
|  |  | **18.9** | | 65.9 | 12.4 | 2.85 | |
|  |  | **18.9** | | 41.6 | 37 | 2.53 | |
|  |  | **25.4** | | 57.6 | 14.5 | 2.49 | |
|  |  | **31.6** | | 33.2 | 33.3 | 1.9 | |
|  |  | **35.9** | | 46 | 15.6 | 2.51 | |
|  | **Astrocyte** | 20.3 | | **22.2** | 55.8 | 1.75 | |
|  |  | 17.2 | | **34.6** | 46.2 | 2.01 | |
|  |  | 33.6 | | **43.1** | 20.7 | 2.52 | |
|  |  | 15.6 | | **47.8** | 34.1 | 2.53 | |
|  |  | 24.2 | | **56.7** | 16.5 | 2.59 | |
|  |  | 19.2 | | **65.5** | 12.3 | 2.99 | |
|  |  | 9.81 | | **66.7** | 20.6 | 2.95 | |
|  |  | 12.9 | | **76.4** | 8.1 | 2.59 | |
|  | **Oligodendrocyte** | 18.2 | | 72.2 | **6.86** | 2.8 | |
|  |  | 23.3 | | 62.7 | **11.2** | 2.87 | |
|  |  | 25 | | 57.3 | **15.1** | 2.56 | |
|  |  | 18.2 | | 61.6 | **17.3** | 2.94 | |
|  |  | 23.7 | | 49.2 | **24.6** | 2.44 | |
|  |  | 20.3 | | 45.5 | **31.8** | 2.5 | |
|  |  | 14.7 | | 37.8 | **45.4** | 2.13 | |
|  |  | 9.77 | | 26.3 | **62.2** | 1.67 | |
|  | **Microglia** | 17.7 | | 29.9 | 51.4 | **1.01** | |
|  |  | 23.8 | | 55.4 | 19.4 | **1.38** | |
|  |  | 17.5 | | 41.9 | 39 | **1.67** | |
|  |  | 24.7 | | 60.9 | 12.3 | **2.01** | |
|  |  | 15 | | 51.2 | 31.4 | **2.47** | |
|  |  | 20.1 | | 64.8 | 12.3 | **2.71** | |
|  |  | 12.7 | | 48.3 | 34.9 | **4.09** | |
|  |  | 21.3 | | 60.6 | 13.5 | **4.57** | |
| For each target cell type the distribution was pre-defined to cover a broad range of biological viable proportions (highlighted in bold). | | | | | | | |

| **Table S5. Evaluation of deconvolution accuracy.** Overall and cell-type specific root-mean-squared error (RMSE) for reference panel, calculated using the leave-one-out approach for three deconvolution algorithms implemented in CellMix package. | | | | | |
| --- | --- | --- | --- | --- | --- |
| **Algorithm** | **Overall** | **Neuron** | **Astrocyte** | **Oligodendrocyte** | **Microglia** |
| **ssNMF^a^** | 0.064 | 0.054 | 0.055 | 0.028 | 0.017 |
| **PSEA^b^** | 0.089 | 0.08 | 0.052 | 0.058 | 0.025 |
| **DSA^c^** | 0.465 | 0.32 | 0.328 | 0.291 | 0.295 |
| **^a^** ssNMF: semi-supervised learning non-negative matrix factorization.  **^b^** PSEA: population-specific expression analysis (also named meanProfile in CellMix implementation).  **^c^** DSA: Digital sorting algorithm. | | | | | |

| **Table S6. Comparison of the cellular proportions estimated using the method PSEA in AD and control brains from the Mayo and Mount Sinai Brain Bank.** | | | | | | | | | |
| --- | --- | --- | --- | --- | --- | --- | --- | --- | --- |
| **Brain Regions** | **Sample Size** | **Neuron** | | **Astrocyte** | | **Oligodendrocyte** | | **Microglia** | |
| **Mayo** | N | Effect | P-value | Effect | P-value | Effect | P-value | Effect | P-value |
| Cerebellum | 119 | -0.04 | 1.05×10^-01^ | 0.06 | 2.09×10^-02^ | -0.02 | 1.53×10^-01^ | 8.79×10^-04^ | 6.53×10^-01^ |
| Temporal Cortex | 119 | -0.17 | 7.1×10^-08^ | 0.23 | 8.43×10^-09^ | -0.07 | 3.1×10^-02^ | 3.91×10^-04^ | 8.67×10^-01^ |
| **Mount Sinai Brain Bank** |  |  |  |  |  |  |  |  |  |
| Anterior Prefrontal Cortex | 184 | -0.04 | 1.75×10^-03^ | 0.06 | 1.02×10^-04^ | -0.01 | 2.68×10^-02^ | -1.81×10^-03^ | 2.76×10^-02^ |
| Superior Temporal Gyrus | 167 | -0.07 | 2.57×10^-06^ | 0.09 | 1.06×10^-06^ | -0.01 | 8.55×10^-02^ | -1.77×10^-03^ | 1.32×10^-01^ |
| Parahippocampal Gyrus | 160 | -0.1 | 2.37×10^-08^ | 0.13 | 2.66×10^-10^ | -0.02 | 2.74×10^-03^ | -1.72×10^-03^ | 2.44×10^-01^ |
| Inferior Frontal Gyrus | 159 | -0.04 | 4.07×10^-03^ | 0.06 | 5.96×10^-04^ | -0.01 | 1.15×10^-01^ | -2.81×10^-03^ | 1.84×10^-02^ |

| **Table S7. Cell-type proportions comparison of subjects diagnosed with Pathological Aging.** The cell-type proportions inferred from RNA-seq data using the ssNMF method. Distribution in Pathological Aging (PA) brains, AD cases and neuropath-free controls are compared using linear regression model. | | | | | | | | | |
| --- | --- | --- | --- | --- | --- | --- | --- | --- | --- |
|  | **Sample Size** | **Neuron** | | **Astrocyte** | | **Oligodendrocyte** | | **Microglia** | |
| **PA vs AD** | N | Effect | P-value | Effect | P-value | Effect | P-value | Effect | P-value |
| Cerebellum | 75 | 0.05 | 6.66×10^-02^ | -0.06 | 3.83×10^-02^ | 0.02 | 1.11×10^-01^ | -0.01 | 2.29×10^-04^ |
| Temporal Cortex | 76 | 0.19 | 2.51×10^-06^ | -0.21 | 1.02×10^-06^ | 0.03 | 5.57×10^-02^ | -0.01 | 5.05×10^-04^ |
| **PA vs Control** |  |  |  |  |  |  |  |  |  |
| Cerebellum | 94 | 0.02 | 5.38×10^-01^ | -0.04 | 1.77×10^-01^ | 0.02 | 1.7×10^-01^ | 4.65×10^-03^ | 1.73×10^-01^ |
| Temporal Cortex | 91 | -0.05 | 1.91×10^-01^ | -0.03 | 4.58×10^-01^ | 0.07 | 2.75×10^-02^ | 5.39×10^-03^ | 1.53×10^-01^ |

| **Table S8. Effect of AD in the neuronal and astrocytic proportions in distinct cerebral cortex areas.** Comparison of the effect that AD has in the cell-type distribution in the four cerebral cortex areas ascertained in the Mount Sinai Study (ANCOVA). We report p-values for the pairwise comparison (upper triangle for neuron and lower triangle for astrocyte). | | | | |
| --- | --- | --- | --- | --- |
|  | **Upper Half - Neuron** | | | |
| **MSBB regions** | **APC** | **STG** | **PHG** | **IFG** |
| **APC^a^** | - | 7.79×10^-04^ | 9.02×10^-03^ | <2.22×10^-16^ |
| **STG^b^** | 4.89×10^-01^ | - | 9.35×10^-01^ | 2.12×10^-11^ |
| **PHG^c^** | 9.90×10^-01^ | 7.06×10^-01^ | - | 8.53×10^-13^ |
| **IFG^d^** | 1.28×10^-10^ | 2.23×10^-06^ | 8.86×10^-08^ | - |
|  | **Lower Half - Astrocyte** | | | |
| **^a^ APC** - Anterior Prefrontal Cortex.  **^b^ STG** - Superior Temporal Gyrus.  **^c^** **PHG** - Parahippocampal Gyrus.  **^d^** **IFG** - Inferior Frontal Gyrus. | | | | |

| **Table S9. Comparison of the cellular proportions estimated using the method PSEA in AD and control brains from the DIAN and Knight-ADRC** | | | | | | | | | |
| --- | --- | --- | --- | --- | --- | --- | --- | --- | --- |
| **Disease Status** | **Sample Size** | **Neuron** | | **Astrocyte** | | **Oligodendrocyte** | | **Microglia** | |
|  | N | Effect | P-value | Effect | P-value | Effect | P-value | Effect | P-value |
| **AD^a^ vs Control** | 122 | -0.1 | 1.41×10^-03^ | 0.12 | 7×10^-05^ | -0.02 | 1.97×10^-01^ | -1.84×10^-03^ | 2.4×10^-01^ |
| **ADAD^b^ vs Control** | 38 | -0.18 | 2.35×10^-06^ | 0.22 | 3.03×10^-10^ | -0.05 | 3.47×10^-02^ | -3.77×10^-03^ | 1.56×10^-04^ |
| **LOAD^c^ vs Control** | 100 | -0.08 | 1.16×10^-02^ | 0.1 | 6.82×10^-04^ | -0.02 | 2.5×10^-01^ | -1.12×10^-03^ | 4.91×10^-01^ |
| **ADAD vs LOAD** | 106 | -0.09 | 9.47×10^-04^ | 0.12 | 1.1×10^-05^ | -0.03 | 6.23×10^-02^ | -1.14×10^-04^ | 9.38×10^-01^ |
| **^a^** AD Includes all of the AD affected subjects from the Knight-ADRC and DIAN studies.  **^b^** ADAD: autosomal dominant AD, carriers of pathogenic mutation in *APP*, *PSEN1* or *PSEN2*.  **^c^** LOAD: late-onset AD patients not carrying any autosomal dominant mutation. | | | | | | | | | |

| **Table S10. Allelic effect of *APOE* ε4 allele in the cellular proportion of brain from AD cases and controls included the Mayo Clinic and Mount Sinai Brain Bank.** The cell-type distributions for all samples were inferred from bulk RNA-seq using the ssNMF method. The allelic effect of *APOE* ε4 was estimated using linear regression model. | | | | | | | | | | |
| --- | --- | --- | --- | --- | --- | --- | --- | --- | --- | --- |
| **Brain Region** | **Sample Size** | **Neuron** | | **Astrocyte** | | **Microglia** | | **Oligodendrocyte** | | |
| **Mayo** | N | Effect | P-value | Effect | P-value | Effect | P-value | Effect | P-value |  |
| Cerebellum | 119 | 0.01 | 4.98×10^-01^ | -7.59×10^-04^ | 9.70×10^-01^ | 3.36×10^-03^ | 1.36×10^-01^ | -0.02 | 4.32×10^-02^ |  |
| Temporal Cortex | 119 | -0.06 | 9.91×10^-03^ | 0.1 | 4.15×10^-04^ | 1.16×10^-04^ | 9.56×10^-01^ | -0.04 | 1.77×10^-02^ |  |
| **Mount Sinai Brain Bank** |  |  |  |  |  |  |  |  |  |  |
| Anterior Prefrontal Cortex | 154 | -0.02 | 1.71×10^-01^ | 0.03 | 2.97×10^-02^ | -1.00×10^-03^ | 2.56×10^-01^ | -0.01 | 1.07×10^-03^ |  |
| Superior Temporal Gyrus | 145 | -0.02 | 1.00×10^-01^ | 0.03 | 6.69×10^-02^ | 3.88×10^-04^ | 7.22×10^-01^ | -0.01 | 2.42×10^-01^ |  |
| Parahippocampal Gyrus | 122 | -0.05 | 4.52×10^-03^ | 0.06 | 7.77×10^-03^ | -4.72×10^-04^ | 6.91×10^-01^ | -4.6×10^-03^ | 4.34×10^-01^ |  |
| Inferior Frontal Gyrus | 135 | -0.02 | 2.35×10^-01^ | 0.03 | 5.23×10^-02^ | -1.57×10^-03^ | 1.55×10^-01^ | -0.01 | 1.48×10^-02^ |  |
| Multi-regions | 556 | -0.03 | 2.75×10^-04^ | 0.04 | 8.06×10^-06^ | -4.33×10^-04^ | 4.31×10^-01^ | -0.01 | 2.42×10^-03^ |  |

| **Table S11. Allelic effect of *APOE* ε4 allele in the cellular proportion of brain restricted to AD cases included the Mayo Clinic and Mount Sinai Brain Bank.** The cell-type distributions for all samples were inferred from bulk RNA-seq using the ssNMF method. The allelic effect of *APOE* ε4 was estimated using linear regression model. | | | | | | | | | |
| --- | --- | --- | --- | --- | --- | --- | --- | --- | --- |
| **Brain Regions** | **Sample Size** | **Neuron** | | **Astrocyte** | | **Microglia** | | **Oligodendrocyte** | |
| **Mayo** | N | Effect | P-value | Effect | P-value | Effect | P-value | Effect | P-value |
| Cerebellum | 50 | 0.05 | 5.70×10^-02^ | -0.04 | 7.26×10^-02^ | 2.94×10^-03^ | 2.14×10^-01^ | -0.01 | 5.09×10^-01^ |
| Temporal Cortex | 52 | -4.54×10^-03^ | 8.39×10^-01^ | 0.01 | 8.06×10^-01^ | 7.10×10^-05^ | 9.73×10^-01^ | -1.67×10^-03^ | 8.45×10^-01^ |
| **Mount Sinai Brain Bank** |  |  |  |  |  |  |  |  |  |
| Anterior Prefrontal Cortex | 64 | -0.01 | 6.46×10^-01^ | 0.02 | 2.65×10^-01^ | 8.72×10^-05^ | 9.43×10^-01^ | -0.01 | 5.11×10^-03^ |
| Superior Temporal Gyrus | 59 | -0.02 | 2.40×10^-01^ | 0.02 | 4.12×10^-01^ | 1.39×10^-03^ | 4.29×10^-01^ | 2.39×10^-03^ | 7.56×10^-01^ |
| Parahippocampal Gyrus | 46 | -0.04 | 5.84×10^-02^ | 0.04 | 8.92×10^-02^ | -1.96×10^-03^ | 3.57×10^-01^ | -1.27×10^-03^ | 8.74×10^-01^ |
| Inferior Frontal Gyrus | 56 | -0.02 | 2.78×10^-01^ | 0.03 | 2.03×10^-01^ | -2.30×10^-03^ | 2.26×10^-01^ | -0.01 | 4.35×10^-01^ |
| Multi-regions | 225 | -0.03 | 1.60×10^-02^ | 0.03 | 1.03×10^-02^ | -2.07×10^-04^ | 8.20×10^-01^ | -3.46×10^-03^ | 3.29×10^-01^ |

**Fig S1** **Principal component analysis of samples included in the reference panel a)** Transcriptome-wide. Genes included in the reference panel **b)** PC1 vs PC2 and **c)** PC3 vs PC4.

**Fig S2 Leave-one-out evaluation of reference panel a)** Gene expression levels (log-transformed) for reference panel. The cell types of the isolated/iPSC-derived samples are color-coded and labeled on the y-axis. **b)** We follow a leave-one-out procedure to obtain the cellular proportion for each of the samples of the reference panel. Cell-type proportions are shown as stacked percentage (red: astrocytes; green: microglia; blue: neuron; purple: oligodendrocyte).

**Fig S3** **Chimeric Library Deconvolution Simulation** Human cell-type specific reference samples were combinationed (N=720) to generate chimeric libraries. Reads were randomly sampled following 32 pre-specified distributions (Neuronal reads contributed between 2 to 36% of reads, astrocytes between 22 to 76%, oligodendrocytes between 6 to 62% and microglia between 1 to 5%). Each chimeric library was quantified and the cellular distribution estimated using digital deconvolution. These estimates were compare to prior distribution.

**Fig S4 Cellular population structure of cell-type specific samples** Cell-type proportions shown as stacked percentage (red: astrocytes; green: microglia; blue: neuron; purple: oligodendrocyte). **a)** iPSC derived cortical neurons (N = 8). **b)** mouse barrel cortex neurons isolated by Translating Ribosome Affinity Purification (TRAP) procedure (N = 24). **c)** iPSC derived microglia (N = 10).

**Fig S5 Microglia and oligodendrocyte proportions inferred from RNA-seq of Mayo Clinic and Mount Sinai Brain Bank (MSBB) studies** Mean microglial (green) and oligodendrocyte (purple) proportion for AD cases and neuropath-free controls (bars indicate the standard deviation). The numbers of subjects are indicated below x-axis.

**Fig S6 Cellular population structure for Alzheimer Disease (AD) and Pathological Aging (PA) subjects included in the Mayo Clinic study** Columns height represent the mean proportions. The numbers of subjects for each group is reported below x-axis.

**Fig S7 Neurons and astrocytes distributions for the brains included in the Mount Sinai Brain Bank stratified by CDR and Braak staging** Neuron (blue) and astrocyte (red) proportions for the plotted against **a)** CDR. **b)** Braak Staging.

**Fig S8 Microglia and oligodendrocyte proportions of DIAN and Knight-ADRC participants** Mean microglia (green) and oligodendrocyte proportions (purple) for the DIAN and Knight-ADRC studies (bars indicate standard deviation). Numbers of subjects for each group reported below x-axis. **a)** Distributions for autosomal dominant AD (ADAD) carriers of pathogenic mutations in *APP, PSEN1 or PSEN2*; Late-onset AD (LOAD); and controls. **b)** Braak Staging and **c)** Clinical dementia rating (CDR) states; **d)** Distributions of sporadic LOAD participants, carriers of *PLD3* and *TREM2* variants and controls.

**Fig S9 Neurons and astrocytes distributions for samples included in the Mayo Clinic and Mount Sinai Brain Bank (MSBB) stratified by *APOE* ε4 allele** Neuronal (blue) and astrocyte (red) proportions. **a)** AD Cases and controls. **b)** Restricted to AD cases

**Fig S10 Comparison of cell proportions estimated from full-depth and down-sampled RNA-seq data** Each sample of the reference panel sample was down-sampled (400,000 reads) and cellular population structure inferred following leave-one-out procedure. Cell-type proportions of the samples inferred using the full-depth RNA-seq data are presented along the X-axis, and along the Y-axis the counterparts inferred using shallow RNA-seq.

**References**

1. Gong T, Hartmann N, Kohane IS, Brinkmann V, Staedtler F, Letzkus M, Bongiovanni S, Szustakowski JD: **Optimal deconvolution of transcriptional profiling data using quadratic programming with application to complex clinical blood samples.** *PLoS One* 2011, **6:**e27156.

2. Zhong Y, Wan YW, Pang K, Chow LM, Liu Z: **Digital sorting of complex tissues for cell type-specific gene expression profiles.** *BMC Bioinformatics* 2013, **14:**89.

3. Gaujoux R, Seoighe C: **Semi-supervised Nonnegative Matrix Factorization for gene expression deconvolution: a case study.** *Infect Genet Evol* 2012, **12:**913-921.

4. Kuhn A, Thu D, Waldvogel HJ, Faull RL, Luthi-Carter R: **Population-specific expression analysis (PSEA) reveals molecular changes in diseased brain.** *Nat Methods* 2011, **8:**945-947.

5. Repsilber D, Kern S, Telaar A, Walzl G, Black GF, Selbig J, Parida SK, Kaufmann SH, Jacobsen M: **Biomarker discovery in heterogeneous tissue samples -taking the in-silico deconfounding approach.** *BMC Bioinformatics* 2010, **11:**27.

6. Cahoy JD, Emery B, Kaushal A, Foo LC, Zamanian JL, Christopherson KS, Xing Y, Lubischer JL, Krieg PA, Krupenko SA, et al: **A transcriptome database for astrocytes, neurons, and oligodendrocytes: a new resource for understanding brain development and function.** *J Neurosci* 2008, **28:**264-278.

7. Holtman IR, Raj DD, Miller JA, Schaafsma W, Yin Z, Brouwer N, Wes PD, Moller T, Orre M, Kamphuis W, et al: **Induction of a common microglia gene expression signature by aging and neurodegenerative conditions: a co-expression meta-analysis.** *Acta Neuropathol Commun* 2015, **3:**31.

8. Zhang Y, Sloan SA, Clarke LE, Caneda C, Plaza CA, Blumenthal PD, Vogel H, Steinberg GK, Edwards MS, Li G, et al: **Purification and Characterization of Progenitor and Mature Human Astrocytes Reveals Transcriptional and Functional Differences with Mouse.** *Neuron* 2016, **89:**37-53.

9. Brennand KJ, Simone A, Jou J, Gelboin-Burkhart C, Tran N, Sangar S, Li Y, Mu Y, Chen G, Yu D, et al: **Modelling schizophrenia using human induced pluripotent stem cells.** *Nature* 2011, **473:**221-225.

10. **AMPAD Knowledge Portal BroadiPSC RNAseq** [<https://www.synapse.org/#!Synapse:syn3607401>]

11. Blake JA, Eppig JT, Kadin JA, Richardson JE, Smith CL, Bult CJ: **Mouse Genome Database (MGD)-2017: community knowledge resource for the laboratory mouse.** *Nucleic Acids Res* 2017, **45:**D723-d729.
